# Calculation of the force field required for nucleus deformation during cell migration through constrictions

**DOI:** 10.1371/journal.pcbi.1008592

**Published:** 2021-05-24

**Authors:** Ian D. Estabrook, Hawa Racine Thiam, Matthieu Piel, Rhoda J. Hawkins

**Affiliations:** 1 Department of Physics and Astronomy, University of Sheffield, Sheffield, United Kingdom; 2 cfaed, TU Dresden, Dresden, Germany; 3 Institut Curie, PSL Research University, CNRS, UMR 144, Paris, France; 4 Cell and Developmental Biology Center, National Heart, Lung, and Blood Institute, National Institutes of Health, Bethesda, Maryland, United States of America; 5 Institut Pierre-Gilles de Gennes, PSL Research University, Paris, France; University of Pennsylvania, UNITED STATES

## Abstract

During cell migration in confinement, the nucleus has to deform for a cell to pass through small constrictions. Such nuclear deformations require significant forces. A direct experimental measure of the deformation force field is extremely challenging. However, experimental images of nuclear shape are relatively easy to obtain. Therefore, here we present a method to calculate predictions of the deformation force field based purely on analysis of experimental images of nuclei before and after deformation. Such an inverse calculation is technically non-trivial and relies on a mechanical model for the nucleus. Here we compare two simple continuum elastic models of a cell nucleus undergoing deformation. In the first, we treat the nucleus as a homogeneous elastic solid and, in the second, as an elastic shell. For each of these models we calculate the force field required to produce the deformation given by experimental images of nuclei in dendritic cells migrating in microchannels with constrictions of controlled dimensions. These microfabricated channels provide a simplified confined environment mimicking that experienced by cells in tissues. Our calculations predict the forces felt by a deforming nucleus as a migrating cell encounters a constriction. Since a direct experimental measure of the deformation force field is very challenging and has not yet been achieved, our numerical approaches can make important predictions motivating further experiments, even though all the parameters are not yet available. We demonstrate the power of our method by showing how it predicts lateral forces corresponding to actin polymerisation around the nucleus, providing evidence for actin generated forces squeezing the sides of the nucleus as it enters a constriction. In addition, the algorithm we have developed could be adapted to analyse experimental images of deformation in other situations.

## Author summary

Many cell types are able to migrate and squeeze through constrictions that are narrower than the cell’s resting radius. For example, both immune cells and metastatic cancer cells change their shape to migrate through small holes in the complex tissue media they move in. During migration the cell nucleus is more difficult to deform than the cell cytoplasm and therefore significant forces are required for a cell to pass through spaces that are smaller than the resting size of the nucleus. Experimental measurements of these forces are extremely challenging but experimental images of nuclear deformation are regularly obtained in many labs. Therefore we present a computational method to analyse experimental images of nuclear deformation to deduce the forces required to produce such deformations. A mechanical model of the nucleus is necessary for this analysis and here we present two different models. Our computational tool enables us to obtain detailed information about forces causing deformation from microscopy images and consequently provide evidence for actin generated forces squeezing the sides of the nucleus as it enters a constriction.

## 1 Introduction

Cell migration is crucial to the function of numerous cell types, for example in tissue development [1], wound healing [2, 3], immune response [4] and cancer metastasis [5, 6]. The importance of cell motility in biology and medicine has motivated a large body of research. In the past, cell migration experiments tended to focus on cells crawling on rigid substrates. However in recent years, an increasing number of investigations are being done on cells in environments that are more relevant to *in vivo* tissues [7]. One aspect of tissue-like environments is that cells are confined. Studies of cells in confinement have shown that cells use different mechanisms to move than used on rigid two dimensional surfaces [8–11]. Here we concern ourselves with the case of cells migrating through micrometer sized constrictions that require significant cellular deformation. Previous work on such microchannel systems by ourselves and others [9, 11–17], indicates that the migration mode is “amoeboid”. The main mechanism in amoeboid motility is actomyosin contraction at the rear of the cell, which pushes it forwards. In addition, actin polymerisation against the channel walls can increase the friction, particularly in cases such as a lack of integrin mediated adhesion [11]. Both actomyosin contraction and actin polymerisation generate cortical flows that generate cell migration [11, 13, 18]. We expect these processes to contribute to nuclear translation and deformation as they do in channels without constrictions. In this work we are particularly interested in what additional mechanisms may be necessary for the nucleus to pass through constrictions smaller than the undeformed size of the nucleus. In this work we calculate the minimum force field necessary to deform the nucleus sufficiently to pass through these constrictions. By comparing our solutions with experimental data on actin intensity, we provide evidence for actin driven compressive deformations of the nucleus.

Eukaryotic cells consist of two main compartments; the cell nucleus containing the genetic material, and the cytoplasm containing the cytoskeleton, cytosol and the remaining organelles. The latter are typically smaller than the constrictions we use here and should not constitute a limitation to cell migration in this context. On the long timescales associated with cell migration, the cytoplasm displays a fluid like response to applied forces. In contrast, the cell nucleus is typically the biggest and stiffest organelle [19] and has a more elastic response [20]. The nucleus is surrounded by the inner and outer nuclear membranes, collectively known as the nuclear envelope [2]. Just inside the inner membrane is a dense intermediate filament network called the nuclear lamina [2], thought to provide the mechanical support for the nucleus and protection for the DNA [21, 22]. Recent work on cell movement through constrictions has highlighted the relevance of the cell nucleus [12, 21–25]. The deformability of the nucleus can be thought of as a rate limiting step in cell migration through constrictions smaller than the size of the undeformed nucleus [26, 27]. In order to pass through such constrictions, a cell must generate sufficient forces to deform the nucleus such that it fits through the constriction. What force magnitudes and directions are necessary to achieve this? Measuring such forces directly is extremely challenging and has not yet been performed experimentally. However, imaging the nucleus shape is now done routinely in many labs, for example by optical imaging of fluorescently labelled nuclei. Here we therefore present a method to analyse such images to obtain predictions of the force field required to deform the nucleus in the way observed. If the mechanical properties of an object are known, the deformation caused by a known applied force can be calculated directly. However, the inverse calculation to find the unknown force field that causes a known deformation is much more difficult. In fact, if only the shape outlines are known, the deformation field and forces cannot be calculated exactly. This is because there is no unique mathematical mapping between two shapes if only the outlines are known. In this work we consequently develop a numerical simulation method to calculate the force field based on physical modelling.

**Fig 1:**
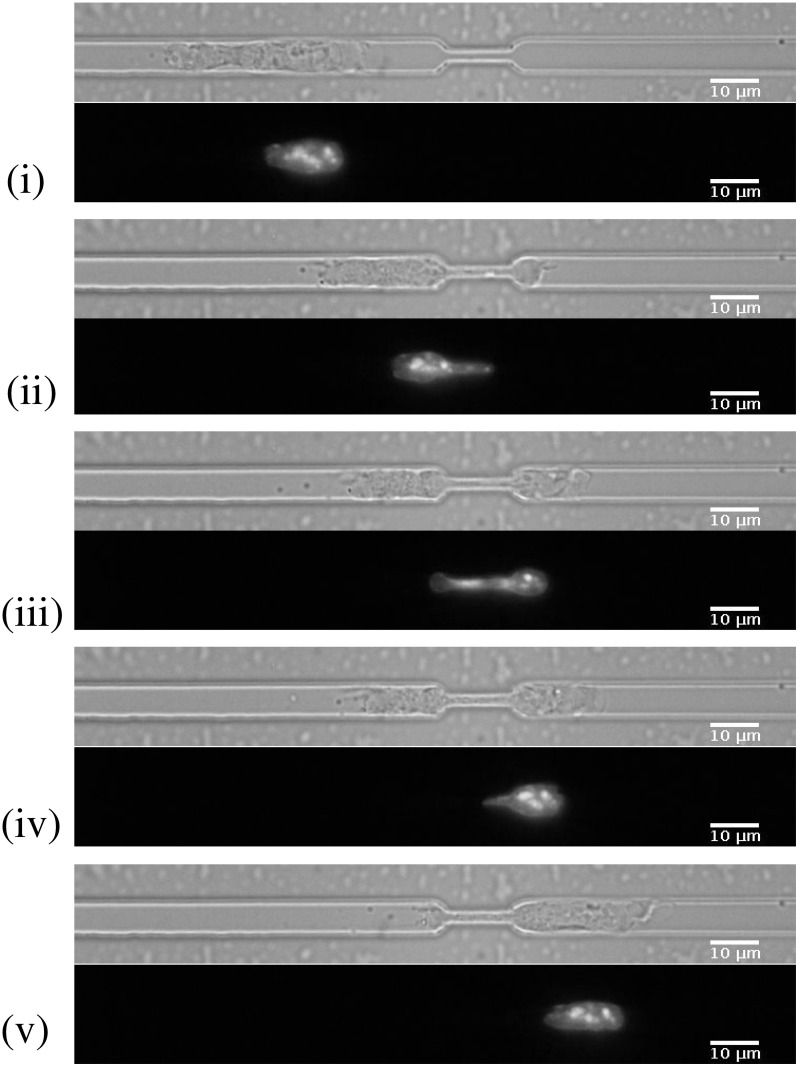
Mouse bone marrow derived dendritic cell passing through a constriction in a microfabricated channel. Specific time points (i)-(v) selected from a time-lapse spinning disk confocal movie. (Top) Transmitted light images of the cell, (Bottom) Fluorescence image of the nucleus of the same cell, stained with Hoechst. Scale bar = 10 *μ*m; constriction width = 2 *μ*m; channel width = 7 *μ*m. The selected time points are; (i) before the constriction, (ii) beginning to enter the constriction, (iii) within the constriction, (iv) exiting the constriction and (v) after the nucleus has fully exited the constriction.

In this study we consider the case of cells migrating through microfabricated channels [28] (see Fig 1). In this setup there are no external flows or chemotactic gradients and the spontaneously motile cells move themselves through the channels, entirely self-generating the required forces. The channels are made of polydimethylsiloxane (PDMS) with a glass bottom through which the microscopy images are taken. All surfaces of the enclosed channels are coated with fibronectin. We choose the height and width of the microchannels to match the cell size in order to effectively constrain cells to one dimensional motion along the long axis of the channel. We design constrictions in the channels such that cells and their nuclei must deform to pass through the constrictions. We obtain sequential images of dendritic cells with the nuclear DNA fluorescently stained as the cells migrate through constrictions in the microchannels. From these images we extract the shape of the nucleus at each time point.

A mechanical model is necessary to deduce the force fields causing the nucleus deformations observed in experiments such as the microchannel experiments we focus on. To find a deformation field between the different nucleus shapes obtained from the experimental images, we use a mechanical model and a simulated annealing approach to find the minimum energy deformation. We then use our mechanical model to calculate the force field required to produce the deformation. This gives us a prediction of the forces generated by the cell to deform the nucleus through constrictions. These predictions are dependent on the mechanical model for the nucleus used. In this work we consider two simple limiting cases of possible models for the nucleus. However, the algorithm we have developed can be used with any mechanical model and therefore is extendable to other models for the nucleus.

To model the nucleus we assume it is an elastic material, governed by the laws of continuum elasticity. We consider two different single component models for the nucleus, namely a homogeneous elastic solid and a thin elastic shell, which treat the extremes of inner material behaviour (see section 2.1). Using such simple models for the nucleus means we only have two elastic parameters. All other quantities are calculated from the experimental input shape coordinates. One of these parameters is given by our measurements of the nuclear volume. The other parameter only affects the force magnitudes with linear scaling. Therefore our simple models are effectively parameter free.

To date a few groups have published models for the nucleus using a variety of approaches. The simplest is a quasi one dimensional model of a linear elastomer [29]. More complex simulation models include a liquid droplet with an elastic interface using the immersed boundary method [30], cellular Potts with an energy cost to deformation [31], finite element analysis of a viscoelastic [32] or poroelastic [33] material and hybrid agent based finite element [34]. The approach closest to ours in spirit is that of [35] who use an elastic model to analytically calculate the energy required to deform the nucleus from an initial spherical shape to an ellipse or a cigar shape. A common feature of all these previous models is that the deformation is imposed by the model, whereas our model calculates the deformation from inputted arbitrary shapes extracted from experimental data. Due to the complexity of experimentally derived shapes we have to calculate the deformation field using numerical simulation. Results from these previous studies indicate under what circumstances a cell is able to enter a constriction. Our model however calculates the force field required for the deformation observed experimentally. In other words, the force exerted on the nucleus is the output of our model not an input. By comparing our predictions of the forces on the nucleus with experimental data on actin density, we provide evidence for a novel mechanism of actin generated forces squeezing the sides of the nucleus to enable it to enter a constriction.

The rest of the article proceeds as follows. We discuss the physical properties of the nucleus in more detail in section 2.1. Then in section 2.2 we introduce the continuum elasticity formalism and present our homogeneous elastic solid and elastic shell models. We describe in section 2.3 the Monte Carlo procedure we use to determine the deformation field from the experimental images. We discuss our results in section 3 and finally conclude with section 3.5.

## 2 Materials and methods

### 2.1 Physical properties of the cell nucleus

In this section we discuss the mechanical properties of the nucleus focusing first on the elasticity of the nucleus and then on its compressibility.

#### 2.1.1 Nucleus elasticity

In general the elasticity of the nucleus might depend on the timescale and on the amplitude of deformation. The nucleus appears elastic on the minutes timescales we are concerned with, as evidenced by Neelam et al. [36] who showed that the nucleus regains its original shape within seconds after removing a deforming force. In the microchannel experiments we analyse, following exit from the constriction the nucleus regains its original shape immediately (within the time resolution of our experiments), implying it is behaving elastically. Returning to the original shape might in general involve active mechanisms such as ion pumps, however such processes are slow and we assume slower than the timescale of the nucleus response seen in our system [37]. The cell nucleus has been seen to display a significantly more elastic response to applied forces than the surrounding cytoplasm in micropipette aspiration experiments on a timescale of minutes [20, 38]. The mechanical properties of the nucleus have also been investigated using atomic force microscopy, in which indentations caused by small applied forces demonstrate that the nucleus is stiffer than surrounding cytoplasm [39]. The nucleus tends to be an order of magnitude stiffer than the rest of the cell in healthy cells [23]. The elastic response of the nucleus is thought to be provided mainly by the nuclear lamina, in particular by lamins A and C [36, 40–43]. The lamina is a ∼ 100 nm thin network of intermediate filaments (lamins) on the inner side of the nuclear envelope [2].

The response of the internal elements of the nucleus is not so clear. Stem cells not expressing lamin A/C show plastic deformation [44]. Nuclei lacking lamins appear viscoelastic if chromatin is tethered to the nuclear membrane but are viscous-like if chromatin is untethered [45]. Recent micromanipulation experiments on isolated nuclei suggest that chromatin dominates the mechanical response to small deformations but the lamin A/C shell dominates the response at large deformations such as those experienced during migration [46]. The timescales (minutes) of our experiment are longer than the ≲ 1 s on which poroelastic behaviour may be observed [33, 47, 48]. Moreover, we find the nuclear volume is constant during our experiments (see section 2.1.2), suggesting that net fluid flow in/out of the nucleus is negligible on the timescales of our experiment. This does not preclude fluid flow within the nucleus on timescales ≲ 1 s, however, on the timescales of nucleus deformations into constrictions observed, the nucleus shows a rapid return to the original nuclear shape after passing through the constriction, indicating that in these experiments, the nucleus behaves predominately elastically. Our two models treat the extremes of inner material behaviour (elastic or completely deformable). In reality we expect the nucleus inner material may be viscoelastic or poroelastic and thus have properties between the two extremes we address in this work.

We characterise the nuclear elasticity by the Young’s modulus and Poisson ratio (see section 2.1.2 for discussion of the latter). We choose a value of 5 kPa for the Young’s modulus of all our nuclei, which is consistent with the reported range of values in the literature [38, 49, 50]. However, since the stress and traction force field are proportional to the Young’s modulus (see Eqs in section 2.2), our results can be easily scaled for different values of the Young’s modulus. The magnitude of the forces is directly proportional to value of the Young’s modulus but the directions of the forces are independent from the value of the Young’s modulus.

#### 2.1.2 Nuclear compressibility

**Fig 2:**
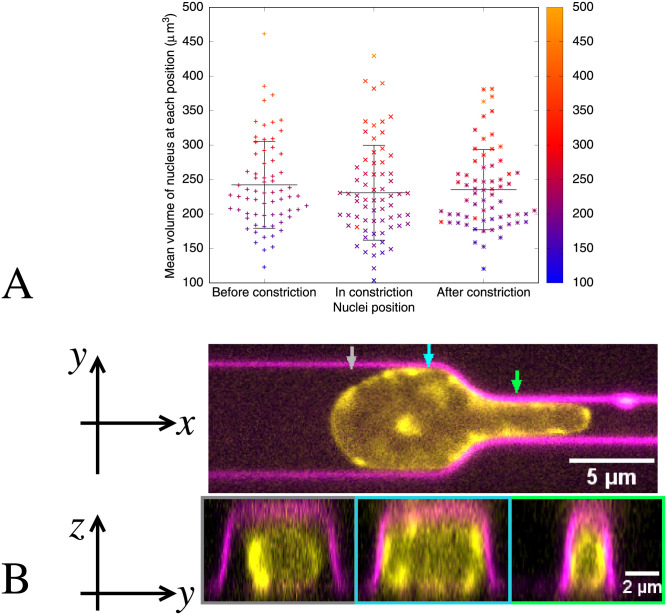
A. The mean volume of each nucleus in *μ*m^3^ before the nucleus enters the constriction, while any part of the nucleus is between the constriction entry and exit, and after the nucleus has exited the constriction. The mean volume of each nucleus is calculated by the mean volume of that nucleus at all available time frames during which the nucleus is before/in/after the constriction. This is a mean of a few time frames since typically there are 10–20 time frames in total for each cell. Each point on the graph represents an individual nucleus with an identifying colour. The colour scale blue to red to orange is the volume of the nucleus before the constriction from small to large. Nuclei in the later positions keep their colour as defined by their volume before the constriction. The fact that the colours of the points stay mostly in the same order shows that the volume of a particular nucleus is roughly constant. The black horizontal lines show the mean volume of nuclei at that position and the vertical black lines indicate the standard deviations. Clearly there is no significant difference in mean nuclear volume as a nucleus travels through the constriction. B. Side view of a nucleus in a channel. Spinning disc confocal image of a dendritic cell with the DNA (in yellow) stained with Hoechst, migrating through a channel with a 2*μ*m wide and 20*μ*m long constriction. Channels are coated with mCherry-PEG (magenta). Top image: top view of the channel. Bottom image: orthogonal/side view of the channel. Arrows (grey, cyan and green) in upper image indicate the slicing regions depicted in the bottom image surrounded by grey, cyan and green rectangles.

To model the nucleus we need to know its compressibility as well as its elasticity. Models often assume incompressibility but some experimental studies have indicated that the nucleus can be compressible [37, 51, 52]. However, whether the nucleus is compressible may depend on timescale, amount of deformation and cell type. Therefore we investigate whether the nucleus undergoes significant volume changes during deformation in our experimental situation. We estimate the volume by measuring the area of the central plane from the experimental images and making assumptions about the geometry in the third dimension. We measure the area (in the plane of the microscope, *x* − *y*) of the nucleus of each cell as it migrates through the constriction (data shown in Appendix A in S1 Text). The nucleus area increases as it goes through the constriction before returning to its pre-entry area, which itself undergoes small fluctuations. To determine the volume we require information about the out of plane direction, *z*. Fig 2B shows the top view in the *x* − *y* plane through the centre of the nucleus (top panel) and the side view at different points along the channel in the *y* − *z* plane (bottom panels). The observed yellow staining indicates that the nucleus fills the channel and the constriction in the *z* direction. We therefore calculate an estimate of each nucleus volume from the known channel geometry. The channel cross section dimensions (width × height) are 7*μ*m × 4.7*μ*m outside the constriction, and 2*μ*m × 3.4*μ*m inside the constriction. We estimate the nucleus volume by calculating the area within and without the constriction and multiplying it by the respective heights. Fig 2A shows the mean volumes of each nucleus before, inside and after the constriction, showing there is no clear volume increase or decrease as nuclei pass through the constriction. There is no statistically significant difference between the mean volumes over all the nuclei before the constriction, (242 ± 63) *μ*m^3^, in the constriction, (231 ± 70) *μ*m^3^, or after the constriction (235 ± 58) *μ*m^3^ (mean ± standard deviation). The 99% confidence interval difference between before and in, before and after and in and after respectively are (−18 < 11 < 41)*μ*m^3^, (−20 < 7.0 < 34)*μ*m^3^ and (−33 < −4.5 < 24)*μ*m^3^ with *t*(68) = 1.0, *p* = 0.85, *t*(68) = 0.67, *p* = 0.75 and *t*(68) = 0.41, *p* = 0.66 respectively.

The change in projected density of DNA, as determined by the fluorescence intensity, is also consistent with the channel height change in the constriction (see Appendix A in S1 Text).

From these measurements we conclude that the nuclei in our experiments do not significantly change volume during deformation through the constrictions. In our models we therefore treat the nucleus as an incompressible material with Poisson ratio *ν* = 0.5.

### 2.2 Continuum elasticity

We treat the nucleus as an elastic object, as assumed for example by [29, 35, 49] (see also discussion in section 2.1.1). We use a continuum approach, justified by our interest in the properties of the nucleus as a whole on length scales much larger than the constituent molecules.

The standard constitutive equation for a continuum elastic solid obeying Hooke’s law is given by [53]

$$\begin{array}{c} \sigma_{ij}=\frac{E}{1+\nu }(\epsilon_{ij}+\frac{\nu }{1-2\nu }\epsilon_{kk}\delta_{ij}) \end{array}$$ (1)

where *σ*_*ij*_ is the stress tensor, *E* is the Young’s modulus, *ν* is the Poisson ratio and *ϵ*_*ij*_ is the strain tensor (*u*_*ij*_ in [53]). The subscripts label tensor elements and *δ*_*ij*_ is the Kronecker delta function. We use the Einstein summation convention for repeated indices. Note that for an incompressible material *ϵ*_*kk*_ = 0. The strain tensor is defined by:

$$\begin{array}{c} \epsilon_{ij}=\frac{1}{2}(\frac{\partial u_{i}}{\partial x_{j}}+\frac{\partial u_{j}}{\partial x_{i}}+\frac{1}{2}\frac{\partial u_{k}}{\partial x_{i}}\frac{\partial u_{k}}{\partial x_{j}}) \end{array}$$ (2)

where *u*_*i*_ is the deformation from a reference configuration and *x*_*i*_ is the position vector. Note that here we keep the second order terms, which are often neglected for small deformations. The deformations we are interested are the sequential deformations between different time points and therefore we take the reference configuration as that of the previous time point. This gives us the energy and forces associated with the deformation due to passing through the constriction, excluding the deformation of a spherical nucleus entering a channel i.e. our starting reference state is not that of a nucleus under zero force but one that is pre-stressed due to the channel confinement.

The free energy density (per unit volume), *f*, of a deformed elastic solid expressed in terms of the strain and stress tensors is given by [53];

$$\begin{array}{c} f=\frac{1}{2}\sigma_{ij}\epsilon_{ij}. \end{array}$$ (3)

The traction force is defined as the external force on a unit area of the surface of a body. For an elastic object it is given by [53];

$$\begin{array}{c} t_{i}=\sigma_{ij}n_{j}, \end{array}$$ (4)

where *n*_*i*_ are the components of the normal to the surface.

In the following we present two elastic models; treating the nucleus as a homogeneous elastic solid (section 2.2.1) and as a thin shell (section 2.2.2).

#### 2.2.1 Homogeneous elastic solid model

We first consider the simplest model of a nucleus as a single homogeneous elastic solid. This assumes the inner nuclear material is elastic on migration timescales and can be thus treated in the same way as the lamina. This is the fully elastic extreme of the models we consider and is useful for comparison to the shell model and to provide the upper limit of the forces required for deformation. Since the strains in our case are not sufficiently small to neglect the nonlinear term we take the full expression given in Eq 2.

To treat the nucleus as an elastic solid, we require the deformation field within the nucleus. Due to the limits in spatial and temporal resolution and lack of clear landmarks, we cannot obtain this directly from the experimental images. We therefore extrapolate the deformation field inside the nucleus from the deformation of the experimentally obtained nuclear outlines. We consider deformations in the centre of mass frame of the nucleus. We assume the centre of mass has zero deformation between any two given images, i.e. the deformation field at (0, 0, 0) is (*u*_*x*_, *u*_*y*_, *u*_*z*_) = (0, 0, 0). We then assume that the deformation field decreases linearly along the radii from each boundary point to zero at the centre of mass. We calculate the centre of mass of a nucleus from its input outline by assuming constant density and using the pixels along the nucleus perimeter to construct a polygon as an approximation of the shape of the nucleus. We then use the standard mathematical result for the centroid of a general 2D polygon, given by [54],

$$\begin{array}{c} \left(x_{c}^{j},y_{c}^{j}\right)=\frac{1}{6A}\sum_{i=1}^{N}\left(\left(x_{i}y_{i+1}-x_{i+1}y_{i}\right)\left(x_{i}+x_{i+1},y_{i}+y_{i+1}\right)\right), \end{array}$$ (5)

where $\left(x_{c}^{j},y_{c}^{j}\right)$ are the coordinates of the centroid of frame *j* of the video, *A* is the area of the polygon, and (*x*_*i*_, *y*_*i*_) are the coordinates of each of the *N* points (pixels) making up the polygon representing the outline of the nucleus. The polygon is a closed loop such that the final point *N* connects directly to the initial point *i* = 1.

To numerically calculate the derivatives in Eq (2) we use finite difference methods (see Appendix B in S1 Text for details).

Fig 2B shows that the cell nucleus completely fills the channel in the *z* direction. Therefore we assume that the nucleus curvature in the *z* direction is zero, both within the channel and inside the constriction. Around the entrance and exit of the constriction where the height is changing there will be a non-zero curvature in the *z* direction. However, this does not affect our calculations since we calculate traction forces in the *x* − *y* plane which are independent from those in the *z* direction. We assume that the images of cell nuclei in the *x* − *y* plane (such as Fig 1) correspond to the central plane of the nucleus, *z* = 0. We use the incompressibility condition to determine the strain component in the *z* direction, *ϵ*_*zz*_ = −(*ϵ*_*xx*_ + *ϵ*_*yy*_), where the strain components *ϵ*_*xx*_ and *ϵ*_*yy*_ are calculated from the deformation fields of the images in the *x* − *y* plane. Since the deformation in the *z* direction, *u*_*z*_, varies only along the channel direction, *x*, the strain components *ϵ*_*yz*_ = *ϵ*_*zy*_ = 0. In the central plane, at *z* = 0, the deformation *u*_*z*_ = 0 and strain components *ϵ*_*xz*_ = *ϵ*_*zx*_ = 0.

Whilst in general a non zero *σ*_*zz*_ stress term exists, the curved surface of the nucleus is defined so that its normal is perpendicular to the *z* direction. Therefore the *σ*_*zz*_ term does not contribute to the traction force on the surface seen in images. The traction force, Eq (4), on the central plane can then be solved based on the two dimensional problem, since the traction over the central plane is unaffected by the surfaces at *z* ≠ 0.

#### 2.2.2 Thin elastic shell model

In this section we present our model of the cell nucleus as a thin elastic shell. We briefly describe the differential geometry needed to calculate the strains here and give further details in Appendix C in S1 Text. We treat the nucleus as a thin elastic shell assuming the inner part of the nucleus contributes no resistance to deformation. This means that in principle the inner medium could change volume, however in our case the observed deformations do not significantly change volume (see section 2.1.2). Our shell model is motivated by the different responses to deformation of different components of the nucleus, as described in section 2.1. Specifically, we treat only the nuclear lamina as having an elastic response to deformation and assume the inner nuclear material provides no resistance to deformation. Therefore we do not need to determine the deformation inside the body, as was required for the solid model in section 2.2.1.

Treating the nuclear lamina as an elastic shell is motivated by the assumption that the elastic response of the nucleus is provided mainly by the nuclear lamina, in particular by lamins A and C [36, 40–43]. We assume the nuclear lamina is a homogeneous elastic material with the same elastic parameters as we used for the whole nucleus in the solid model (section 2.2.1). The amount of any pre-stress in the shell or excess area prior to entry to the constriction has not been established experimentally. Therefore, for simplicity, we assume there is no pre-stress nor excess area.

The nuclear lamina is only about ∼ 100 nm thick [2, 55], which is thin compared to even the smallest constriction width of 2 *μ*m. We therefore use a thin shell approximation in the calculations below. We follow the Love-Kirchhoff thin plate theory [56] as used by Berthoumieux et al. [57] for their model of the cell actomyosin cortex. This thin shell approximation assumes the thickness of the shell remains constant and the normal to the surface remains normal after deformation. This means that the only non-zero strains are those in tangential directions. It is therefore convenient to calculate the strains in the normal and tangential coordinate basis, before transforming back to Cartesian coordinates.

**Fig 3:**
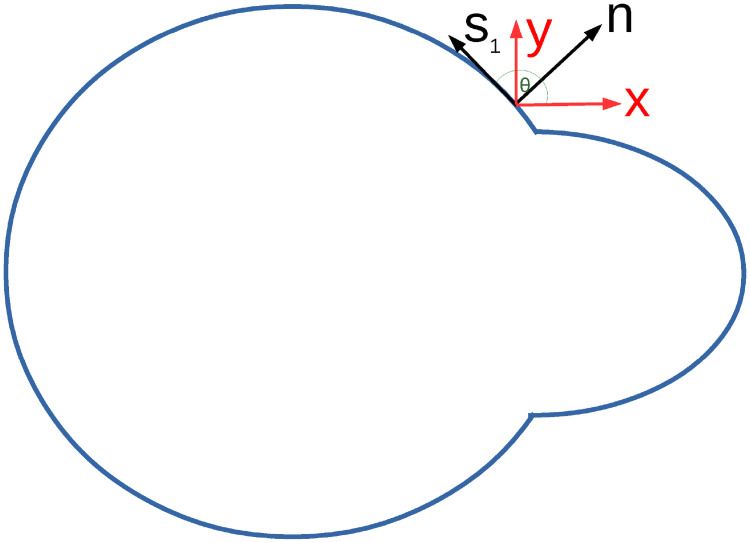
A cartoon of a deforming nucleus in 2D showing the coordinate basis (*n*, *s*_1_, *s*_2_) used for the shell model compared to the Cartesian coordinate system used in the 2D images. NB *s*_2_ is out of the plane.

As drawn in Fig 3, we define the normal and tangential coordinates as (*n*, *s*_1_, *s*_2_) where *s*_1_ is along the tangent to the surface in the plane of the images (with unit vector ${\hat{s}}_{1}$), *s*_2_ is along the surface in the out of plane direction (with unit vector ${\hat{s}}_{2}$) and *n* is along the outward normal to the surface (with unit vector $\hat{n}={\hat{s}}_{1}\times {\hat{s}}_{2}/\left|{\hat{s}}_{1}\times {\hat{s}}_{2}\right|$). We assume the shape is symmetric in the out of plane direction and therefore that the normal, $\hat{n}$, is in the *x*-*y* plane and the tangent ${\hat{s}}_{2}$ is along the $\hat{z}$ direction. This means our coordinate basis is orthonormal and therefore the metric tensor is the identity matrix and all Christoffel symbols are zero so the derivatives of the basis vectors along the surface are given by the curvature tensor, *C*_*ij*_, i.e. $\partial_{i}{\hat{s}}_{j}=C_{ij}\;\hat{n}$, where the curvature tensor $C_{ij}=(\frac{\partial {\hat{s}}_{j}}{\partial s_{i}})\cdot \hat{n}=-(\frac{\partial \hat{n}}{\partial s_{i}})\cdot {\hat{s}}_{j}$. We assume that the surface of the nucleus has zero curvature in the out of plane direction (apart from at the constriction entrance and exit), as motivated by the assumption that the nucleus is symmetric and fills the channel at all times, as in section 2.1.2. As discussed in section 2.2.1 the surfaces at *z* ≠ 0 do not affect the traction forces in the *x* − *y* plane. In this case the only non zero term in the curvature is $C_{s_{1}s_{1}}$ which is calculated numerically from the image data.

The deformations of surface points are initially calculated in Cartesian coordinates. We transform these to our normal and tangential basis using standard anticlockwise rotation matrices *R*_*ϕ*_ as ***u***(*n*, *s*_1_, *s*_2_) = *R*_*ϕ*_ ***u***(*x*, *y*, *z*) where *ϕ* is the angle between $\hat{n}$ and $\hat{x}$ in our 2D images. Using this basis, we can calculate the strain, Eq 2, and then transform back to Cartesian coordinates; $u\left(x,y,z\right)=R_{\phi }^{T}u\left(n,s_{1},s_{2}\right)$. We then calculate the stress and traction from Eqs (1) and (4) as for the solid model.

Note that, as for the solid model, we assume the traction forces on the central plane are unaffected by the edges at finite *z* values and therefore we do not consider the part of the shape with components to the normal in the $\pm \hat{z}$ direction.

### 2.3 Calculation of the deformation field

We calculate the deformation field in the central *x*-*y* plane from the 2D experimental images at consecutive time points. We first find a list of coordinates defining the outline of each image using the image analysis software ImageJ [58]. We convert the image to binary using the set threshold tool and then use the analyse particles tool to obtain the nuclear outline curves at each time point. We choose the threshold to distinguish between the class of bright pixels that are in the nucleus and those that are in the noise far from the nucleus. We do this by plotting the histogram of number of pixels against intensity and setting the threshold value to minimise the intra-class variance (Otsu’s method). Finally, we use the spline fitting and interpolation tools to obtain a list of boundary coordinates, spaced one pixel apart. The list of points on this curve is then used as the input to our algorithm described below.

#### 2.3.1 Averaging of different images

To reduce the effects of noise in the data, we averaged data for many cells (between 55 and 71 with sufficient resolution) see Fig 2 for the nuclear volumes of these cells. To save computational time we averaged the input shapes and then calculated the forces required for these averaged nucleus deformations. We averaged the nuclei according to position relative to the entrance of the constriction. The points we chose are: the nucleus prior to entry (the frame before the nucleus begins deforming in to the constriction), the nucleus entering (chosen as the first frame where the nucleus begins deforming into the channel), the nucleus entirely within the channel (the first frame where the rear of the nucleus completely passes the constriction entrance), the nucleus exiting (the first frame where the front of the nucleus passes the constriction exit) and the nucleus after leaving the constriction (the first frame where the rear of the nucleus has moved past the constriction exit). The average outline is obtained by first finding the geometrical centre of each individual outline and aligning the images with their centroid at the origin. Note that in our model the centre of mass is at the same point as the geometrical centre (the centroid). We then draw spokes radially outwards from the centroid to the outline at fixed angular values, every $\frac{2\pi }{n}$, where *n* is the average number of pixels in the outlines. Then the coordinates of each spoke at the same angle for each shape are summed up and divided by the number of input shapes to give the final average nucleus shape for that time point.

#### 2.3.2 Alignment of nuclear images to remove translation

To define the deformation field between two images of cell nuclei under motion, we need to choose a point to register the images. Our aim is to remove the translational motion of the nuclei, so that the forces we calculate are only those causing the shape change of the nuclei. Therefore for the solid model we set the centre of mass of the initial shape to have zero deformation and we assume deformations at the surface of the nuclei linearly drop to zero at the centre of mass. However, unless the deformations are symmetric, the centroid of the target deformed shape will not be at the same point as the centroid of the initial shape. Therefore, we cannot, *a priori* assume the images should be aligned with the nuclei centroids coinciding. When the nucleus starts to enter the constriction the rear moves forwards very little compared to the leading edge. However, we cannot *a priori* assume the images should be aligned with the rear of the nuclei coincident.

The correct alignment point is likely to lie somewhere between the two limiting cases of the centre of masses or the rear/front points. The limits on both the spatial and temporal resolution of the nuclei in the images prevent us from directly identifying the point of zero deformation from a given video. Therefore, to estimate the translation free relative alignment of the nuclei, we measure the change in the front and rear positions of each nuclei between time frames. We set the centroid of the initial shape to be at the origin. We then shift the target shape along the *x* axis a distance proportional to the measured relative change in the rear and front positions. See Appendix D in S1 Text for details.

#### 2.3.3 Simulated annealing

In this section we describe the Monte Carlo simulated annealing method we use to determine the deformation field between two images of a cell nucleus. The algorithm we have developed reads in a list of coordinates describing the perimeter line of a 2D image for each time step. In our case these coordinates are obtained from experimental images of nuclei, an example of which is given in Fig 1B. However, the same algorithm could be used for other elastically deforming objects. Our aim is to determine the deformation field between two images of the cell nucleus boundary. However, mathematically there is no unique mapping between two such images. We assume that the mapping describing the physical deformation is the one that minimises the free energy of deformation, given by Eq (3). To calculate this energy minimised deformation field, we use a simulated annealing approach, as described below.

In order to determine the deformation field between two images of cell nuclei, the nucleus must be tracked from image to image. One common method to determining the deformation fields between images is by following known ‘landmarks’ in the images [59, 60]. A landmark is a recognisable area that can be used to determine a local deformation field. The global deformation field is then extrapolated from the local deformation of landmarks. However, due to the complex nature of the nucleus and the limited resolution currently available, there are no consistently reliably identifiable landmarks in the Hoechst stained images of the nucleus described above. A different approach is needed in order to determine the deformation field from images lacking clear landmarks. We therefore determine the deformation field through an energy minimisation simulated annealing routine. That is, we seek the mapping that minimises the free energy of the deformation, given by Eq (3), and we assume that this mapping describes the physical deformation the nuclei undergoes. We calculate the deformation field for the outline of the nucleus from each image, as described below.

Consider two input image boundaries at consecutive time points. The nucleus deforms from the first image shape to the second. We assume that boundary points maintain sequential order during any deformation. The first image we will refer to as the initial image and the second as the target image. We first choose an arbitrary mapping between the initial and target image boundaries to obtain an approximation to the deformation field. We will then refine this using a simulated annealing approach to find the deformation field that minimises the free energy. To define the arbitrary mapping, we first order the list of boundary pixel coordinates for each image by angular position and then remesh the initial boundary so it has the same number of points as the target boundary, at equally spaced angles. For the solid model, this remeshing of the boundary points also defines the inner radial mesh. We calculate the free energy of this arbitrary starting deformation using Eq (3).

We now perturb the deformation field to find configurations with lower free energy. We randomly choose one of the initial pixels and perturb the position of its mapped point on the target image. We move the chosen pixel a tenth of the way towards one of the two neighbours, with the direction chosen at random. We calculate the free energy of this new configuration. If the free energy of deformation decreases, the new deformation location is recorded as a new minimal energy configuration. If the free energy increases the energy by an amount Δ*E*, the configuration is kept if $\exp(-\frac{\Delta E}{k_{B}T})$ is greater than a randomly generated number between 0 and 1. If not, then the change is discarded, and a new perturbation performed. Perturbations are continued until a minimum energy is reached. The temperature *T* is gradually reduced during the simulated annealing procedure from an initial value such that 20% of moves are accepted, as in standard simulated annealing procedures, which are described in more detail in [61, 62]. In principle this procedure could fail if there was more than one energy minimum. However, the procedure works well for small deformations within the validity of the elasticity model we use. Our method would fail for deformations beyond the elastic limit (for example a ruptured shell) or for deformations causing the surface to bend back upon itself causing multi-valued surface points at a particular angle. We find it works well for the types of deformation we study here.

## 3 Results and discussion

There is now a large body of evidence indicating that nucleus is the main physical hindrance to cell migration through tissues [23]. It is therefore crucial to understand how cells deform the nucleus, especially professionally migrating cells such as dendritic cells. In this section we present our results for the traction force field on the nucleus calculated from the deformation field of average shape changes calculated as in section 2.3.1, using the aligning estimated as described in section 2.3.2 and the simulated annealing method described in section 2.3.3. We present and compare results for both our homogeneous elastic solid (section 2.2.1) and thin elastic shell (section 2.2.2) models.

### 3.1 Solid model results

**Fig 4:**
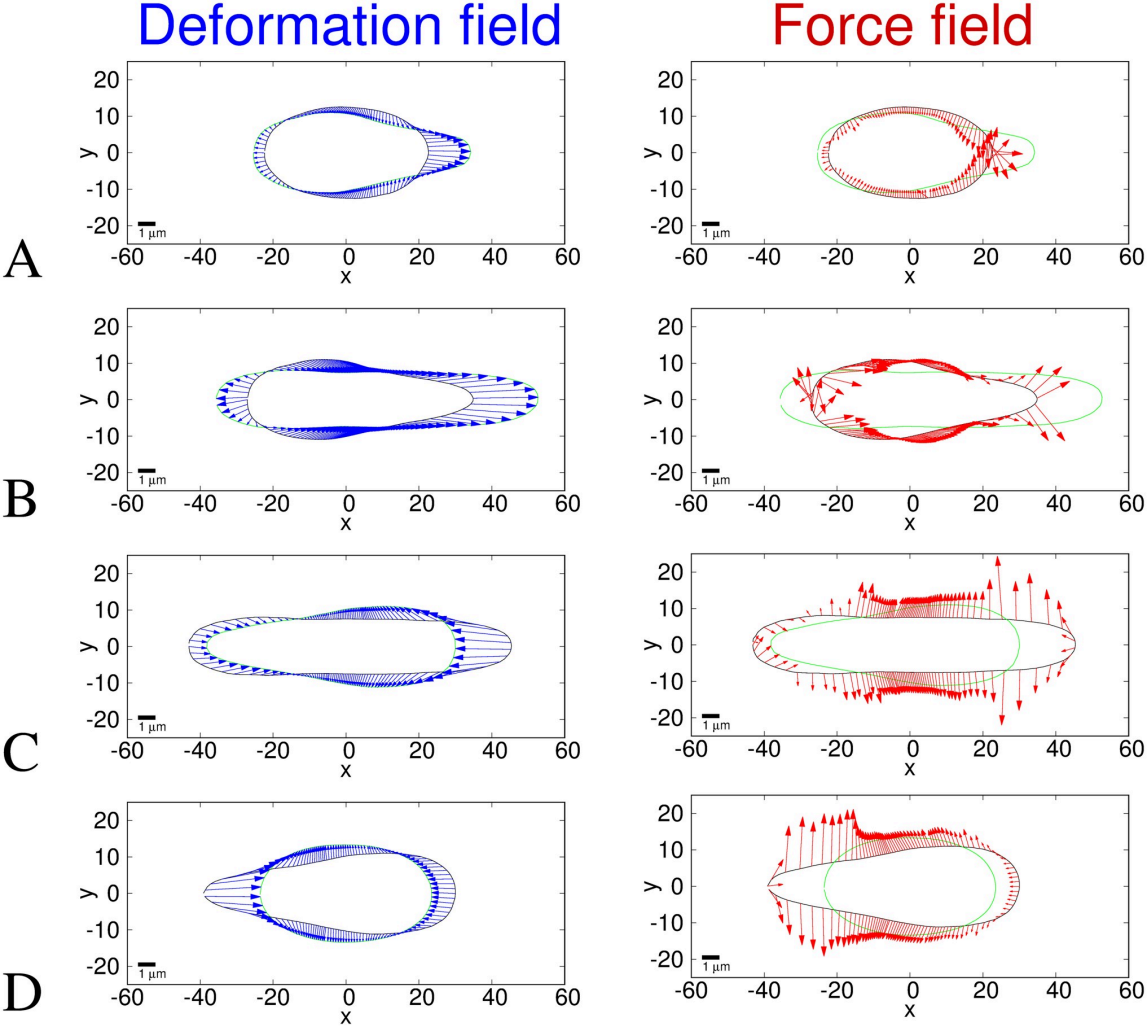
Deformation (left) and traction force (right) fields for the solid model. The axes show pixel numbers where each pixel is 0.215*μ*m and scale bars show 1*μ*m. The black outline is the initial shape and the green outline is the target deformed shape. Blue arrows represent the final deformation field found between the images and red arrows represent the traction force direction and magnitude, with each arrow scaled such that one unit of length on the axes represents a traction force of 250 Pa. The traction force is calculated using a Young’s modulus of *E* = 5 kPa and assuming that the nucleus behaves as an incompressible elastic solid with a Poisson ratio *ν* = 0.5. A. Initial shape is the average of 71 nuclei before the constriction and the target shape is the average of 56 nuclei beginning to enter the constriction. B. Initial shape is the average of 56 nuclei entering the constriction and the target shape is the average of 71 nuclei within the constriction. C. Initial shape is the average of 71 nuclei within the constriction and the target shape is the average of 55 nuclei as they begin exiting the constriction. D Initial shape is the average of 55 nuclei exiting the constriction, to the average shape of 71 nuclei after they have fully exited the constriction. Since the shapes are averages they do not correlate directly to any single nucleus, however experimental images of a typical nucleus are shown for comparison in Fig 1. The target shape is moved −1.7, −7.3, 0.15, 0.0 pixels along the *x* direction, from the centroid aligned position for A-D respectively (see Appendix D in S1 Text).

**Fig 5:**
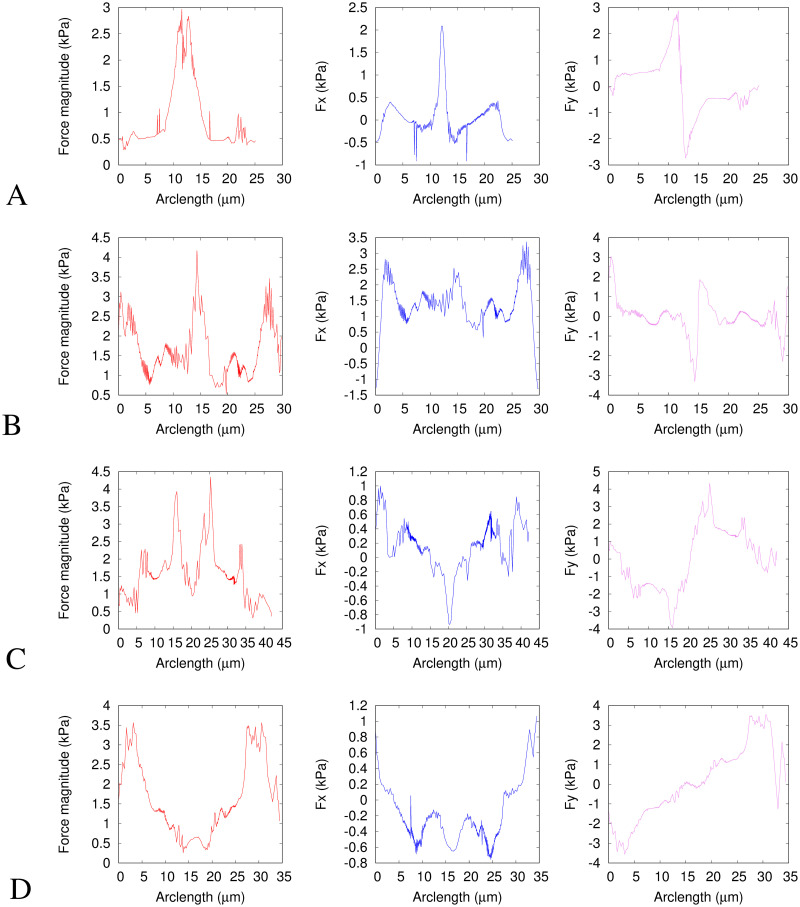
Traction force fields for the solid model. The first column (red curves) shows the traction force magnitudes (the same data as the red arrows in Fig 4). The second column (blue curves) shows the *x* component of the traction forces (along the channel). The third column (magenta curves) shows the *y* component of the traction forces (perpendicular to the channel). These are all plotted against the arclength in micrometers. The direction of the arclength coordinate *s*_1_ is anticlockwise as shown in Fig 3. *s*_1_ = 0 corresponds to *θ* = −*π* and *y* = 0 at the rear of the nucleus (*x* most negative) in Fig 4. *s*_1_ then increases anticlockwise around the negative *y* region to the front (*x* most positive) then around the positive *y* region to rear again. The traction force is calculated using a Young’s modulus of *E* = 5 kPa and assuming that the nucleus behaves as an incompressible elastic solid with a Poisson ratio *ν* = 0.5. The subfigure rows correspond to the same times as in Fig 4, i.e. A. before to entering constriction, B. entering to within constriction, C. within to exiting constriction and D exiting to after constriction.

Fig 4 shows the solid model deformations and traction force fields between the average nuclear shapes at the time points chosen with respect to the constriction: A. before to entering, B. entering to fully inside, C. fully in to exiting and D. exiting to fully after. Fig 5 shows the same force data as in Fig 4 plotted in a different way. The first column shows the force magnitudes against arclength around the nucleus shape. The second and third columns show the *x* and *y* components of the forces against arclength. Note that in our elastic model, the Young’s modulus, *E*, linearly scales the magnitudes of the traction forces without affecting their directions nor distributions.

For clarity in Fig 4, we only show the deformation field and traction forces on the surface of the nucleus. However, the inner part of the nucleus in this solid model also deforms and experiences stress. As explained in section 2.2.1, we assume the deformation scales linearly from its value at the surface to zero at the centre of mass of the nucleus. This results in the radial components of the stress being constant along the radial direction.

In Figs 4A and 5A, the nucleus begins to enter the constriction. The resulting traction force in Fig 4A shows inward compression on the nucleus everywhere except close to the rear and front of the nucleus where the forces are outwards. This is to be expected since the width of the nucleus needs to narrow to enter the constriction. The largest magnitude forces (∼ 4 kPa) are at the leading edge, seen clearly as the central peak in Fig 5A. We also show the free energy density per unit volume, Eq 3, against the arclength in in Appendix E in S1 Text. The traction force as the nucleus goes from entering to fully inside the constriction, shown in Fig 4B has a positive *x* component almost everywhere (shown in Fig 5B middle column). This is predictable, given that the front of the nucleus moves more compared to the rear of the nucleus during this step than in any of the other time steps (see Appendix D in S1 Text). Of particular interest is the increase in force magnitudes around the sides of the nucleus in addition to those at the front and back, seen as the appearance of secondary peaks at *s*_1_ ∼ 8*μ*m and *s*_1_ ∼ 22*μ*m in Fig 5B. The role of these forces is to further push the sides of the nucleus inwards to squeeze it into the constriction. We discuss a potential biological mechanism for generating these lateral forces in section 3.3. Fig 4C shows the traction force as the nucleus changes shape from inside the constriction to beginning to exit. Apart from the rear and the very front, the traction forces are outwards as the shape widens on exiting the channel. These outward forces are larger in magnitude near the front than the rear, due to the larger deformation of the front than the rear as the nucleus exits the constriction. These forces near the front are visible as the largest peaks in the first and third columns in Fig 5C. Also noticeable, particularly in Fig 4C, is that the energy minimisation process results in points making up the surface being closer together in the centre than at the ends. This is due to the fact that the centre of mass of the initial shape undergoes zero deformation. The final figure panel, Fig 4D shows the traction force on the nucleus as it changes shape from the average during exit of the constriction, to the average when the nucleus has fully left the constriction. The average shapes used are notably similar to the shapes taken before the constriction and on entering, as we would expect from an object displaying elastic behaviour. The magnitude of the force is now significantly larger at the rear than at the front (see Fig 5D), which coincides with the rear of the nucleus now deforming more to regain the shape prior to the constriction, whilst the front of the nucleus has already left the constriction so deforms less at this point.

Large compressive lateral inward forces with components perpendicular to the channel axis are clearly seen in Figs 4A, 4B, 5A and 5B, exerted on the nucleus when it enters the constriction. This is quite different from the axial force expected from the common view that cells push the nucleus through the constriction by actomyosin contractility at the back building up a pressure difference between the back and front [7, 35, 63]. The competing idea that the cell pulls the nucleus from the front (using either actomyosin contraction and connection to the substrate at the front, or microtubules connecting the nucleus to the cell front) would also generate axial forces. If we consider the cell generates a force parallel to the channel axis as it does in both push and pull models and in cell migration, the normal physical reaction force (due to Newton’s third law at the physically confining sloped walls, see Fig 1) at the entrance to the constriction will have a component inwards perpendicular to the channel axis. However, the inward force magnitudes we calculate are larger than that expected for the component due to the passive physical reaction of the sloped walls to a forward migratory force (maximum half the forward force for a 45° angle). In Figs 4A and 5A middle column we can see some of these lateral forces are even directed backwards (negative *x*) and therefore cannot be caused only by passive physical reaction forces equal and opposite to a forward cell generated force. From Figs 4B, 4C, 5B and 5C it is clear that inward compressive forces on the nucleus also occur inside the constriction perpendicular to the walls and channel axis. These perpendicular forces cannot be a passive physical reaction opposed to a cell-generated force parallel to the axis of motion. We estimate from [35] that for the nuclei in our experiments to pass through the constriction with a purely forward force it would need to be an order of magnitude larger than the forces we calculate. This therefore implies an additional active force generated by the cell to compress the nucleus on entry to and in the constriction. Thiam et al. [12] proposed that an additional force might be important, with actin polymerisation compressing the sides of the nucleus. Whilst Thiam et al. [12] gave clear evidence that this actin was important for nuclear passage, they provided no direct proof of mechanism, nor an explanation why it would be important. Here we show directly that active forces are deforming the nucleus from the sides. In section 3.3 we compare our results with the actin measured by Thiam et al. [12] providing the first evidence that this lateral actin is exerting forces deforming the nucleus.

The outward forces in Fig 4C and 4D are indicative of an elastic response of the nucleus favouring a return to its original shape once the nucleus leaves the constriction. These outward forces perpendicular to the channel walls seen in Figs 4C, 4D, 5C and 5D third column correspond well with the forces implied by the reflection interference contrast microscopy (RICM) measurement by Thiam et al. [12]. They found by RICM that the only parts of the cells in close contact with the channel walls are the cell rear and inside the constriction. Fig 4C and 4D suggest that close contact with the channel walls would be seen when the nucleus is in, and exiting from, the constriction.

### 3.2 Shell model results

**Fig 6:**
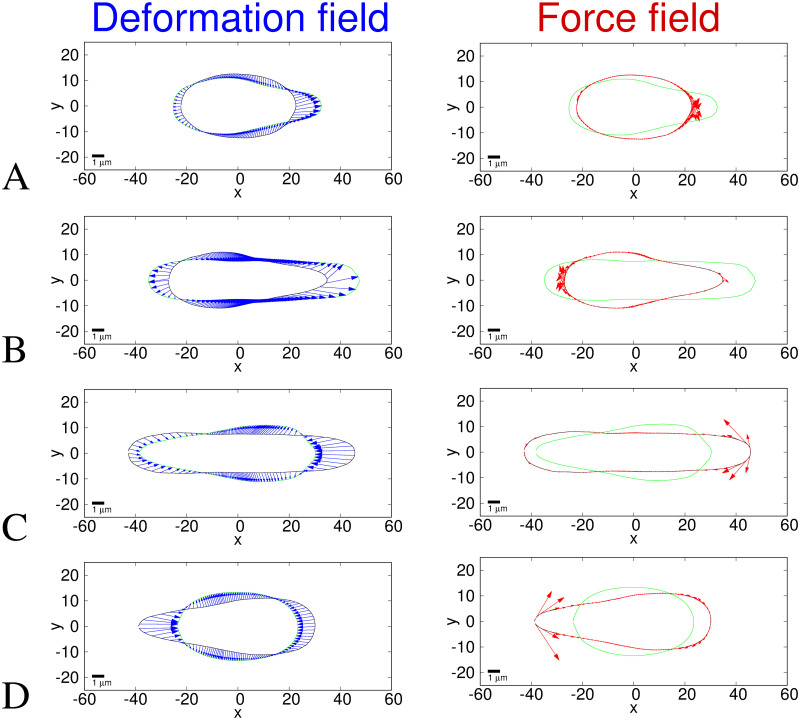
Deformation (left) and traction force (right) fields for the shell model. The axes show pixel numbers where each pixel is 0.215*μ*m. The black outline is the initial shape and the green outline is the target deformed shape. Blue arrows represent the final deformation field found between the images and red arrows represent the traction force direction and magnitude, with each arrow scaled such that one unit of length on the axes represents a traction force of 250 Pa. The traction force is calculated using *E* = 5 kPa and assuming that the nucleus behaves as an incompressible elastic shell with a Poisson ratio *ν* = 0.5. Average shapes the same as in Fig 4, i.e. A. before to entering constriction, B. entering to within constriction, C. within to exiting constriction and D exiting to after constriction. Since the shapes are averages they do not correlate directly to any single nucleus, however experimental images of a typical nucleus are shown for comparison in Fig 1. As in Fig 4, the target shape is moved −1.7, −7.3, 0.15, 0.0 pixels along the *x* direction, from the centroid aligned position for A-D respectively (see Appendix D in S1 Text). A discussion is provided in the main text of the differences between the deformation field and traction forces between the shell model (shown in this figure) and the solid model (shown in Fig 4).

**Fig 7:**
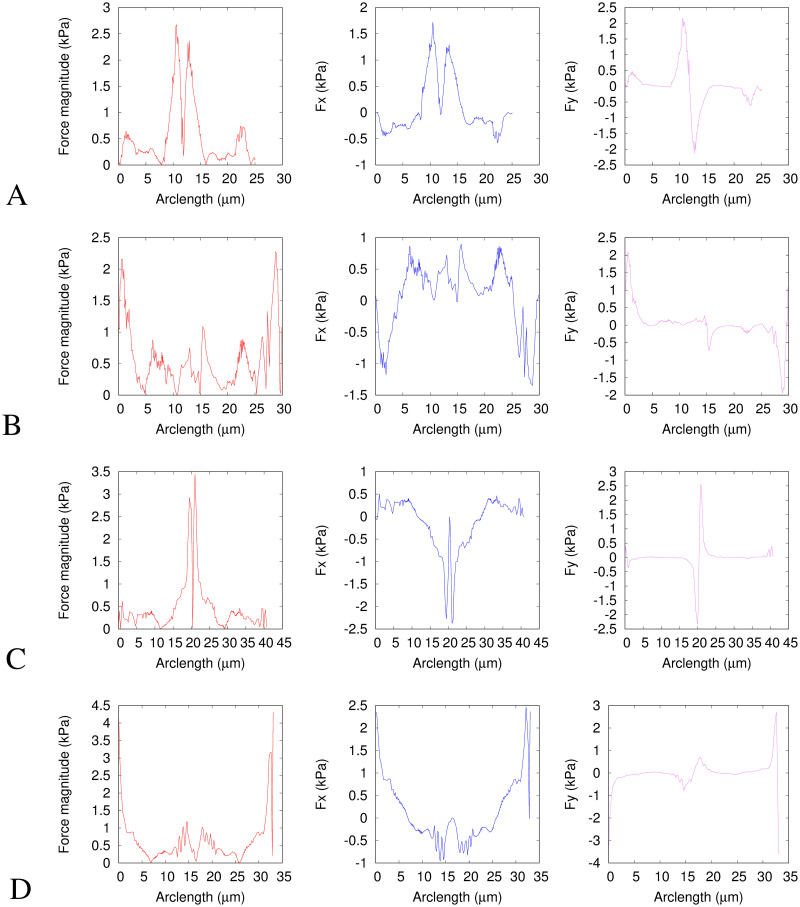
Traction force fields for the shell model. The first column (red curves) shows the traction force magnitudes (the same data as the red arrows in Fig 6). The second column (blue curves) shows the *x* component of the traction forces (along the channel). The third column (magenta curves) shows the *y* component of the traction forces (perpendicular to the channel). These are all plotted against the arclength in micrometers. The direction of the arclength coordinate *s*_1_ is anticlockwise as shown in Fig 3. *s*_1_ = 0 corresponds to *θ* = −*π* and *y* = 0 at the rear of the nucleus (*x* most negative) in Fig 6. *s*_1_ then increases anticlockwise around the negative *y* region to the front (*x* most positive) then around the positive *y* region to rear again. The traction force is calculated using a Young’s modulus of *E* = 5 kPa and assuming that the nucleus behaves as an incompressible elastic solid with a Poisson ratio *ν* = 0.5. The subfigure rows correspond to the same times as in Fig 6, i.e. A. before to entering constriction, B. entering to within constriction, C. within to exiting constriction and D. exiting to after constriction. A discussion is provided in the main text of the differences between the traction forces between the shell model (shown in this figure) and the solid model (shown in Fig 5).

Figs 6 and 7 show the deformation and traction force fields calculated using the shell model for the same time points and average nuclear shapes as shown in Figs 4 and 5. We also show the free energy density per unit volume, Eq 3, against the arclength in Appendix E in S1 Text. The deformation fields in Fig 6 are slightly different from those in Fig 4 due to the model dependence of the deformation energy and therefore the minimisation procedure. In the solid model (Fig 4), points on the target shape are positioned to minimise radial distance whereas in the shell model (Fig 6), the deformation minimises the number of points in regions of high curvature. This is due to the fact that in the shell model the strain is dependant on the curvature due to the derivatives of the basis vectors (see section 2.2.2). Therefore, in the high curvature regions the energy minimisation results in larger deformations between neighbouring points, the cost of which is outweighed by minimising the energy cost due to curvature. This physical effect means the high curvature regions are more stretched and therefore more likely to rupture. We have not included the possibility of rupture in our model but one could consider rupture occurring above a threshold tension.

All the traction forces in Fig 6 are parallel to the surface. This is because in the thin shell approximation the normal component of the strain is zero and, due to the incompressibility condition, a zero normal strain leads to a zero normal stress. Therefore the strain and traction force are purely tangential. In general, the magnitudes of the traction forces in the shell model (mean 0.5 kPa) shown in Figs 6 and 7 are smaller than for the equivalent elastic solid model (mean 1.5 kPa) shown in Figs 4 and 5. This is expected given that for the shell model the inner material has no mechanical resistance so the force required to deform a thin elastic shell is less than that required to deform a solid elastic object.

Of particular interest is the traction force in Figs 6C and 7C which shows large extensile traction forces at the leading tip when the nucleus is at its most elongated inside and exiting the constriction. These forces are suggestive of the forces required to break the lamina [12] and induce nuclear envelope rupture as observed at the front tip of nuclei in small constrictions [21, 22]. Note we do not expect the large forces at the rear seen in Figs 6D and 7D to also cause rupture since these forces occur at the time point when the rear of the nucleus exits from the constriction so the nucleus is then free to reform a less confined shape thus preventing further rupture. However, if the nucleus does not rupture at the tip when the tip exits the constriction, the large tensile forces at the back when the rear exits the constriction might cause rupture, as seen in about a quarter of cases in [21].

### 3.3 Correlation with actin density

**Fig 8:**
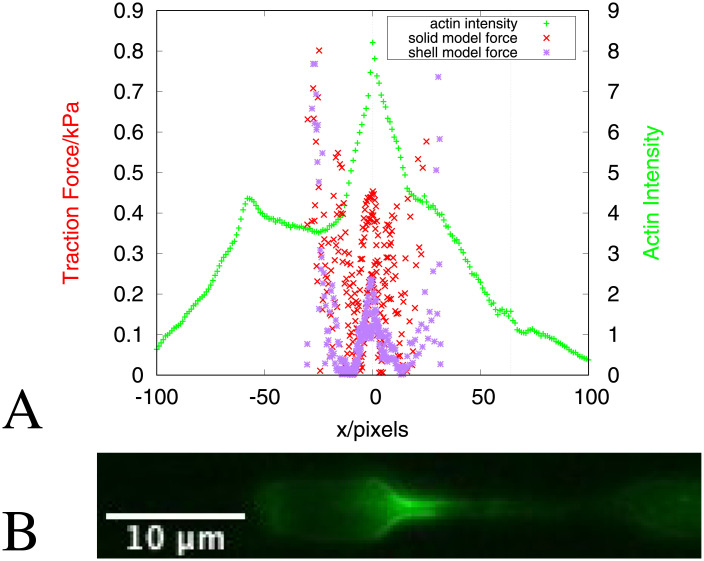
A. Average actin (LifeAct-GFP fluorescence) intensity (green + points) at the time point when the nucleus is entering into the constriction (right hand *y*-axis). The actin intensity is the mean intensity over the width of the channel at each pixel position. This is then renormalised by the average intensity for each cell and aligned with the start of the constriction at *x* = 0. The average is taken over 83 cells. The red × points are the absolute values of the *y* components of the traction force from the solid model (Fig 4B) and the purple ✳ points are the absolute values of the *y* components of the traction force from shell model (Fig 6B). The vertical dotted lines indicate the start and end positions of the constriction. B. Representative experimental image showing the actin intensity.

The lateral inward forces shown in Fig 4A and 4B are suggestive of forces correlated with the Arp2/3 mediated actin assembly around the nucleus when it is in the constriction reported by Thiam et al. [12]. For cells migrating in channels that confine the cell but are larger than the size of the nucleus, no peak in actin density around the nucleus is seen. These cells move due to force generation mechanisms (e.g. actomyosin contractility) that are independent from the Arp2/3 dependent actin polymerisation around the nucleus seen when the nucleus enters a constriction. When Thiam et al. [12] inhibited the actin nucleator Arp2/3 using the drug CK666 this significantly reduced the percentage of nuclei passing through constrictions. Thiam et al also found that low doses of latrunculin depolymerising actin had a similar effect, as did depleting the Wave2 subunit Hem1. This clearly shows that Arp2/3 mediated actin polymerisation is necessary for nuclei to pass through the constrictions. Theoretical modelling is necessary to elucidate how such actin could enable nuclear passage. Hawkins et al [11] showed that, as well as actomyosin contractility generating force along the channel, actin polymerisation against the channel walls can also result in cytoplasmic flow along the *x* direction. In the current context of our studies on the nucleus, actin polymerisation against channel walls at the sides of the nucleus would result in lateral forces on the nucleus pushing inwards causing nuclear deformation instead of cytoplasmic flow. The calculations we present in this manuscript show that such lateral forces are required for the minimum energy nuclear deformation through the constriction. Interestingly the free energy density values we calculate (shown in Appendix E in S1 Text) are of order kJ/m^3^, which is equivalent to fJ/*μ*m^3^, and is equivalent to the energy provided by about one ATP molecule per (10 nm)^2^ of surface area (assuming a depth of 1*μ*m). This is similar to what one would expect for the density of actin filaments polymerising against the surface. This is consistent with the idea that actin polymerisation generates the force required. To see the extent to which our theoretical calculations support the empirical conclusions from the experiments published in Thiam et al. [12], we compare the observed intensity of actin with our force fields. Fig 8 shows the average actin intensity profile along the channel at the point when the nucleus is entering into the constriction. For comparison the traction force magnitudes from the solid model (Figs 4B and 5B) and the shell model (Figs 6B and 7B) are plotted on the same graph. The large forces at the front and rear of the nucleus in Fig 8 are expected from the motility mechanisms (e.g. actomyosin contractility) generating a higher pressure at the back compared to the front. Whilst the pressure gradient generated by such mechanisms is along the *x* axis, pressure always acts perpendicular to the surface and therefore will have some *y* components due to the curved shape of the nucleus. In our comparison between the actin density and traction forces on the nucleus we are not interested in the front or rear of the nucleus but in the sides of the nucleus, since the actin intensity peak is around the sides of the nucleus. Of particular interest therefore is the lateral forces around the sides of the nucleus as it enters the constriction corresponding to the central peak in actin intensity around the beginning of the constriction. The peak of elevated forces at the sides of the nucleus is not expected from a pressure gradient along the channel and therefore this requires an alternative explanation. The corresponding peak in the actin density provides such an explanation. This strongly suggests that the increase in actin around the nucleus at the entrance to the constriction contributes to generating the force necessary to deform the nucleus to pass it through the constriction. By this we therefore provide the first evidence that lateral actin exerts force deforming the nucleus from the sides. This lateral force at the sides of the nucleus in the constriction is necessary for the nucleus to pass through such constrictions. Models such as [33, 35] which assume axial forces only (either pushing or pulling along the axis) predict a threshold constriction size, below which the nucleus would not be able to pass through. With the lateral forces our model predicts however the nucleus is able to pass through smaller constrictions. In addition, the magnitudes of the forces we predict are lower than that required for a model with axial forces only. Thiam et al [12] show that lamin A/C depleted cells are able to pass through constrictions without lateral actin. Lamin A/C depletion softens the nucleus by disrupting the lamina and therefore the elastic shell of the nucleus. Without its elastic shell, we expect the nucleus can pass through the constrictions with only axial forces because it is behaving more like a fluid.

To further validate the correlation between our predicted lateral forces and the actin density we analysed an example of nuclei that fail to pass through the constriction (see Appendix F in S1 Text). This shows no peak in actin density around the start of the constriction. There are large forces at the tip of the nucleus as it attempts to enter the constriction but these are not sufficiently squeezing the nucleus from the sides to enable entry, as is the case for nuclei that successfully pass through the constriction.

### 3.4 Comparison between models

The two limiting cases of models we have studied here both show results indicative of experimental observations. In particular, the normal lateral forces predicted by the solid model correlate with actin density and RICM data from Thiam et al. [12] and the large extensile forces at the leading tip predicted by the shell model correlate with the tip rupture observed by Thiam et al., Denais et al. and Raab et al. [12, 21, 22]. We therefore expect that aspects of both models are important in nuclear deformation and that the actual viscoelastic properties of the nucleus lie between the two simple limiting elastic models we have presented here. The method and algorithm we present here for analysing images of nuclear deformation can be easily used with any chosen input model for the nucleus.

The main difference between the results from the two different models we have presented here is that the shell model forces are all parallel to the nuclear surface whereas the solid model has large normal forces. This difference is clearly seen in the direction of the arrows in Figs 4 and 6. As expected, the force magnitudes are quantitatively smaller in the shell model compared to the solid model (means 0.5 kPa and 1.5 kPa respectively). This is because our simple shell model has an interior presenting no mechanical resistance. The solid model is the opposite extreme with the interior presenting the same amount of mechanical resistance as the outer shell. However, apart from these differences in magnitude and directions, the distribution of these forces over the nuclear surface for these two extreme model limits is remarkably similar. This can be seen most easily by comparing the profiles plotted in Fig 5 with those in Fig 7. Whilst the magnitudes are different, the form of the profiles is similar i.e. at which points along the nucleus the forces are larger or smaller. This suggests that the exact mechanical model used for the nucleus is not crucially important for the force distribution since the general features of the force field profiles are similar for these two extreme models. This is partly because viscous aspects of nuclear mechanics are not important on the timescale of cell migration through a constriction.

For a more realistic model, with nuclear mechanical properties in between these two extreme models, we would expect the distribution of forces to be similar to what we see for both our models but that the magnitudes would be scaled between those we predict i.e. a mean between 0.5 kPa and 1.5 kPa. Importantly, we would expect that the directions of the forces would be between those of our solid model (predominantly normal) and those of our shell model (parallel to the nuclear surface).

### 3.5 Conclusions

We have presented a method for calculating the force acting over the surface of a cell nucleus causing the deformation seen between two images, using a continuum elasticity model. We have used a Monte Carlo simulated annealing simulation method to define the deformation field between two images. We then calculated the traction force causing the observed deformation of averages of the nuclei of cells migrating through a constriction in a confined channel. We have presented two limiting case models for the nucleus as a homogeneous elastic solid and as a thin elastic shell. Since the most realistic model is likely to lie between these extremes, we expect the directions and magnitudes of forces to be between the two cases we have calculated. However, we find that the form of the profile of forces over the nuclear surface is similar in both extreme models.

The predictions we have made of the details of the force fields required to deform a nucleus through a constriction can be used to assess models of force generation mechanisms used by cells to force their nucleus through small constrictions. For example, the forces in the positive *x* direction at the rear of the nucleus, shown in Figs 4B–4D and 6D, are consistent with what would be expected from actomyosin contraction at the rear of the cell generating an increased pressure behind the nucleus pushing it forwards [11, 13, 18]. The forces at the front of the nucleus may also be generated by such an actomyosin contractility generated pressure gradient or by pulling forces at the front of the cell, for example force exerted on the nuclear surface by molecular motors as they move along cytoskeletal filaments. By using our model, we were able to determine that the compressive forces perpendicular to the direction of motion around the sides of the nucleus seen in Fig 4 correlate with the Arp2/3 mediated perinuclear actin network assembly observed by Thiam et al. [12] at the constriction entrance. We therefore conclude that actin polymerisation is correlated with lateral forces around the nucleus that are required to squeeze it into constrictions that are smaller than the threshold size passable with axial forces alone. We expect that the observed actin polymerisation generates forces to push the nucleus inwards in a similar way to that described for the cytoplasm in Hawkins et al. [11]. However, the correlation could be due to actin being induced to polymerise where the forces are the strongest. Our algorithm can be used to quantitatively analyse future experiments to further investigate such mechanisms used to generate nuclear deformation.

Our computational model is not restricted to nuclei within channels and could be applied to any two dimensional images of deforming nuclei, if appropriate assumptions are made. Furthermore our algorithm could be adapted to analyse images of other elastic objects deforming. Our code can be obtained freely on GitLab https://gitlab.com/nucleus-deformation-traction.

## Supporting information

S1 Text Appendices. A. DNA density and Nucleus area. B. Numerical calculation of the strain tensor. C. Differential geometry of surfaces D. Alignment of initial and target images. E. Strain energy F. Example of nuclear failure to pass through the constriction.
(PDF)
