# Supplementary material for: Calculation of the force field required for nucleus deformation during cell migration through constrictions
Source: PLoS Comput Biol. 2021 May 24;17(5):e1008592. doi: 10.1371/journal.pcbi.1008592 (PMC8177636; doi:10.1371/journal.pcbi.1008592)
Supplement: S1 Text — A. DNA density and Nucleus area. B. Numerical calculation of the strain tensor. C. Differential geometry of surfaces D. Alignment of initial and target images. E. Strain energy F. Example of nuclear failure to pass through the constriction. (PDF) [file pcbi.1008592.s001.pdf]

Calculation of the force field required for nucleus deformation during cell migration through constrictions
Supporting information

Ian D. Estabrook[1,2], Hawa Racine Thiam[3,4], Matthieu Piel[3,5], and Rhoda J. Hawkins[1,*]

[1]Department of Physics and Astronomy, University of Sheffield, Sheffield, United Kingdom
[2]cfaed, TU Dresden, Dresden, Germany
[3]Institut Curie, PSL Research University, CNRS, UMR 144, Paris, France
[4]Cell and Developmental Biology Center, National Heart, Lung, and Blood Institute, National Institutes of Health, Bethesda, Maryland, United States
[5]Institut Pierre-Gilles de Gennes, PSL Research University, Paris, France
[*]rhoda.hawkins@physics.org

May 1, 2021

A DNA density and Nucleus area

Another method to determine whether there is any volume change of the nucleus within the channels is to observe the density of DNA within the cell. As DNA is the most dense component within the nucleus, observing how the density of the DNA changes with position in the channel could indicate if there are any significant volume changes. If the nucleus does increase in volume, the average density of materials within the nucleus, including the DNA should decrease and vice versa if the nucleus is compressed. Both density changes and height changes in the out of plane direction should change the observed florescence intensity of the DNA. Therefore, if we observe a change in fluorescence intensity by more than the change in the dimensions of the channel in to the constriction, that would be an indication of overall nuclear volume change.

In order to measure the average intensity of fluorescing pixels, we need to remove the background of the images. We did this using the existing software within imageJ [1], which uses a "rolling ball" method, in order to remove the background of images [2]. However, as in the example shown in [2], this typically leaves the background as pixels with small, but non zero values of fluorescence. In order to accurately count the number of fluorescing pixels, these must not be counted when computing the mean intensity value. To do this, we calculate the average intensity of all the pixels in a given image.

We then calculate the mean value and standard deviation of the fluorescence of the pixels. Then we decrease the intensity of each image by an amount given by $I_{new} = I_{old} - \tilde{I} - a\sigma$, where I_{new} is the intensity with the background removed, I_{old} is the intensity with background, $\tilde{I}$ is the mean intensity of all pixels, σ is the standard deviation of the intensities and a is a value that is dependent on the image. The value of a is increased in increments of 0.1 from 0, until the number of fluorescing pixels matches that of the area of the curves around the nuclei, which we had previously drawn using the threshold, spline fitting and interpolation tools within imageJ, for each given image and frame.

The change in DNA intensity between the nuclear region inside and outside the constriction of $60 - 70\%$ is consistent with the change in height of the channel from 5μm outside of the constriction to 3.4μm within the constriction, i.e. the constriction is approximately 70% of the height of the channel.

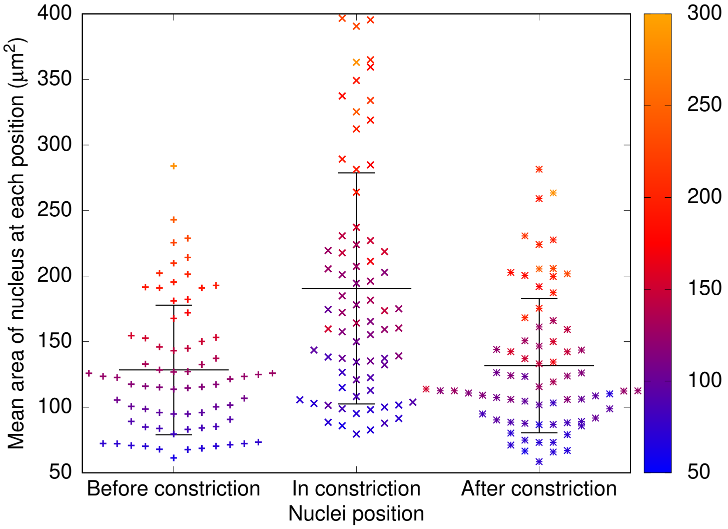

Fig A.1. The mean area of each nucleus in μm^2 before the nucleus enters the constriction, while any part of the nucleus is between the constriction entry and exit, and after the nucleus has exited the constriction. The mean area of each nucleus is calculated by the mean area of that nucleus at all available time frames during which the nucleus is before/in/after the constriction. This is a mean of a few time frames since typically there are 10-20 time frames in total for each cell. Each point on the graph represents an individual nucleus with an identifying colour. The colour scale blue to red to orange is the area of the nucleus before the constriction from small to large. Nuclei in the later positions keep their colour as defined by their area before the constriction. The black horizontal lines show the mean area of nuclei at that position and the vertical black lines indicate the standard deviations.

The change in area shown in Fig A.1 is also consistent with the change in height of the channel from 5μm outside of the constriction to 3.4μm within the constriction. Multiplying this area by the height gives us the volume shown in Fig 2A in the main text. We therefore conclude that there is no significant volume change of the nuclei as they travel through the constrictions.

B Numerical calculation of the strain tensor

In order to accommodate the boundary conditions given by solid model assumption of a deformation field decreasing lineally along the radial direction, we calculate the derivatives in this model using polar coordinates in the 2D plane seen in images. However, the coordinates of the pixels in the experimental images are known in Cartesian coordinates. Therefore a transformation between these two coordinate systems is necessary.

The polar form of the strain and Eq (2) in the main text can be written using

$$\frac{\partial u_i}{\partial x_j} = \frac{\partial u_i}{\partial r}\frac{\partial r}{\partial x_j} + \frac{\partial u_i}{\partial \theta}\frac{\partial \theta}{\partial x_j}, \tag{1}$$

where we can use the standard Polar-Cartesian relations to evaluate the following terms;

$$\frac{\partial r}{\partial x} = \frac{x}{r}, \qquad \frac{\partial r}{\partial y} = \frac{y}{r}, \qquad \frac{\partial \theta}{\partial x} = \frac{-y}{r^2} \quad \text{and} \quad \frac{\partial \theta}{\partial y} = \frac{x}{r^2}. \tag{2}$$

Substituting these into Eq (1) gives the polar form of the strain tensor.

Numerically, we calculate these derivatives using finite central difference forms of numerical derivatives on a mesh. The input pixels give the mesh points along the perimeter. To define the radial mesh, we draw concentric rings with shapes similar to the perimeter but of decreasing size at pixel sized intervals along the radii. We denote these mesh points using the indices n,m to label the points along the perimeter and radii respectively. The finite central difference numerical derivatives are given by,

$$\begin{aligned}
\frac{\partial u_x(x_{n,m}, y_{n,m})}{\partial r} =& \frac{u_x(x_{n,m+1}, y_{n,m+1}) - u_x(x_{n,m-1}, y_{n,m-1})}{r_{n,m+1} - r_{n,m-1}}, \\
\frac{\partial u_y(x_{n,m}, y_{n,m})}{\partial r} =& \frac{u_x(x_{n,m+1}, y_{n,m+1}) - u_x(x_{n,m-1}, y_{n,m-1})}{r_{n,m+1} - r_{n,m-1}}, \\
\frac{\partial u_x(x_{n,m}, y_{n,m})}{\partial \theta} =& \Big(R_{n+1} u_x(x_{n+1,m}, y_{n+1,m})\Delta\theta_n - R_{n-1} u_x(x_{n-1,m}, y_{n-1,m})\Delta\theta_{n+1} \\
&+ u_x(x_{n,m}, y_{n,m})((\Delta\theta_{n+1})^2 - (\Delta\theta_n)^2)\Big) \Big/ \Big(\Delta\theta_{n+1}\Delta\theta_n(\Delta\theta_n + \Delta\theta_{n+1})\Big), \\
\frac{\partial u_y(x_{n,m}, y_{n,m})}{\partial \theta} =& \Big(R_{n+1} u_y(x_{n+1,m}, y_{n+1,m})\Delta\theta_n - R_{n-1} u_y(x_{n-1,m}, y_{n-1,m})\Delta\theta_{n+1} \\
&+ u_y(x_{n,m}, y_{n,m})((\Delta\theta_{n+1})^2 - (\Delta\theta_n)^2)\Big) \Big/ \Big(\Delta\theta_{n+1}\Delta\theta_n(\Delta\theta_n + \Delta\theta_{n+1})\Big),
\end{aligned}$$

where R_{n+1} is the ratio of the radius at perimeter point $n+1$ to the radius at perimeter point n and $\Delta\theta_{n+1}$ is the angle between these points. Similar expressions are used for the innermost and outermost shapes, but replaced with forward/backward finite difference methods respectively. The factors of R_n in the final two equations are included to scale for small variations in the radius between points. These equations, together with the standard relations between Cartesian and polar coordinates allow the strains to be numerically evaluated from Eq (2) in the main text in the Cartesian coordinate basis directly.

To calculate derivatives of the deformation field in the solid model we need to define the deformation at the inner mesh points along the concentric rings inside the perimeter. Consider point n on the outer perimeter ($m = M$) with coordinates $(x_{n,M}, y_{n,M})$ and position vector from the origin to the point defined by $\boldsymbol{r}_{n,M} = (x_{n,M}, y_{n,M})$. The corresponding point on an inner ring is defined as the point where $\boldsymbol{r}_{n,M}$ crosses the inner ring at $\boldsymbol{r}_{n,m}$ where $m < M$ denotes the inner rings. The deformation at perimeter point n, M is $(u_x(x_{n,M}, y_{n,M}), u_y(x_{n,M}, y_{n,M}))$. We assume zero deformation at the origin linearly increasing radially to the deformation at the perimeter. This leads to the assumption that the deformation at the corresponding point on an inner ring is in the same direction as that at point n, M but with a linearly reduced magnitude. i.e. the deformation at the corresponding point n on inner ring m is given by:

$$(u_x^{\text{inner}}(x_{n,m}, y_{n,m}), u_y^{\text{inner}}(x_{n,m}, y_{n,m})) = \frac{|\boldsymbol{r}_{n,m}^{\text{inner}}|}{|\boldsymbol{r}_{n,M}|}(u_x(x_{n,M}, y_{n,M}), u_y(x_{n,M}, y_{n,M})) \quad (3)$$

where $|\boldsymbol{r}_{n,m}^{\text{inner}}|$ is the distance from the origin to the inner ring point n, m corresponding to point n, M on the perimeter. To calculate the derivatives of these deformations numerically at the outer limits we use a backwards difference method.

C Differential geometry of surfaces

In order to generally describe the more complicated unknown surface in the general tangent and normal coordinate basis, and calculate values along the surface, we first describe the general form of the derivatives using differential geometry. The general forms involve the curvatures of the surface and the Christoffel symbols of the surface, and an analytic method to calculate the derivatives is given below.

Briefly, a surface $\boldsymbol{X}(n, s_1, s_2)$ described by two tangential directions s_1, s_2 and the normal direction n has an associated metric tensor given by the derivatives of the surface along each of the directions at each point,

$$g_{ij} = \left(\frac{\partial X(n, s_1, s_2)}{\partial x_i} \cdot \frac{\partial X(n, s_1, s_2)}{\partial x_j}\right), \quad (4)$$

where x_i represents the tangent directions along the surface. For a positively oriented surface, where by definition when travelling along the curve describing the surface, the interior of the curve is on the left, the outwards normal to the surface is then given by

$$\mathbf{n} = \mathbf{e_{s_1}} \times \mathbf{e_{s_2}} = \frac{\partial X(n, s_1, s_2)}{\partial s_1} \times \frac{\partial X(n, s_1, s_2)}{\partial s_2}. \quad (5)$$

The Christoffel symbols are written in terms of the metric tensor as

$$\Gamma_{ij}^k = \frac{1}{2} g^{kl} \left(\frac{\partial g_{il}}{\partial x_j} + \frac{\partial g_{jl}}{\partial x_i} - \frac{\partial g_{ij}}{\partial x_l}\right). \quad (6)$$

Similarly, the curvature of the surface, measured as the rate of change of the normal

direction along the surface can be expressed as a tensor, C_{ij}.

A thin shell surface can be written as a function of only the two tangent directions, $\boldsymbol{X}(n, s_1, s_2)$, and so the metric tensor is a 2x2 matrix, with the components s_1 and s_2 representing two tangent directions along the surface

$$\mathbf{g} = \begin{pmatrix} e_{s1} \cdot e_{s1} & e_{s1} \cdot e_{s2} \\ e_{s1} \cdot e_{s2} & e_{s2} \cdot e_{s2} \end{pmatrix}. \tag{7}$$

The derivatives along the surface of the basis vectors are then given in terms of the curvature and metric as

$$\frac{\partial \mathbf{e_j}}{\partial x^i} = C_{ij}\mathbf{n} + \Gamma^k_{ij}\mathbf{e_k}. \tag{8}$$

Using these relations, we can describe any thin shell surface, for instance those seen in images of the nucleus. Because the images considered are only in two dimensions, an analytical approach is used to describe the out of plane direction s_2, while the in plane images provide s_1 from the outline. The normal direction is assumed outwards and in the XY plane seen in images, and so can be determined purely from the s_1 tangent vector. As the surface is flat in the out of plane direction, the vector in the out of plane direction is easily defined as a unit length vector parallel to the z axis. As such the metric tensor is the identity and the Christoffel symbols are all zero, leaving only the curvature terms in the shell model of this particular out of plane direction shape. However, we include the full differential geometry in the code to allow for use with other, more complicated, shapes beyond the scope of this paper.

D Alignment of initial and target images

The deformation free point is likely to lie somewhere between the two limiting cases described in section 2.3.2. in the main text. For pure translation the deformation would be zero and the centroid alignment would be the translation free alignment. However in our case there is deformation as well as translation and the centroid alignment is only correct if the deformation is symmetric around the centroid. We need to remove the translation part only such that the shapes are aligned at the point of zero deformation. The reason that the centroid alignment is not necessarily the correct alignment is due to the fact that we calculate the centroid of the 2D images rather than the 3D volume and therefore we need a correction to this alignment. However the limits on both the spatial and temporal resolution of the nuclei in the images prevent the point of zero deformation from being identified directly from any given series of images.

In order to estimate the location where the deformed nucleus should be placed relative to the undeformed nucleus, we measured how the position of the front and rear of the nucleus changed between frames. Measurements of the change in the front, ΔF, and rear, ΔR, position of each nucleus were taken between frames at each of the five positions (before the constriction, entering in to the constriction, inside the constriction, leaving the constriction and after the constriction).

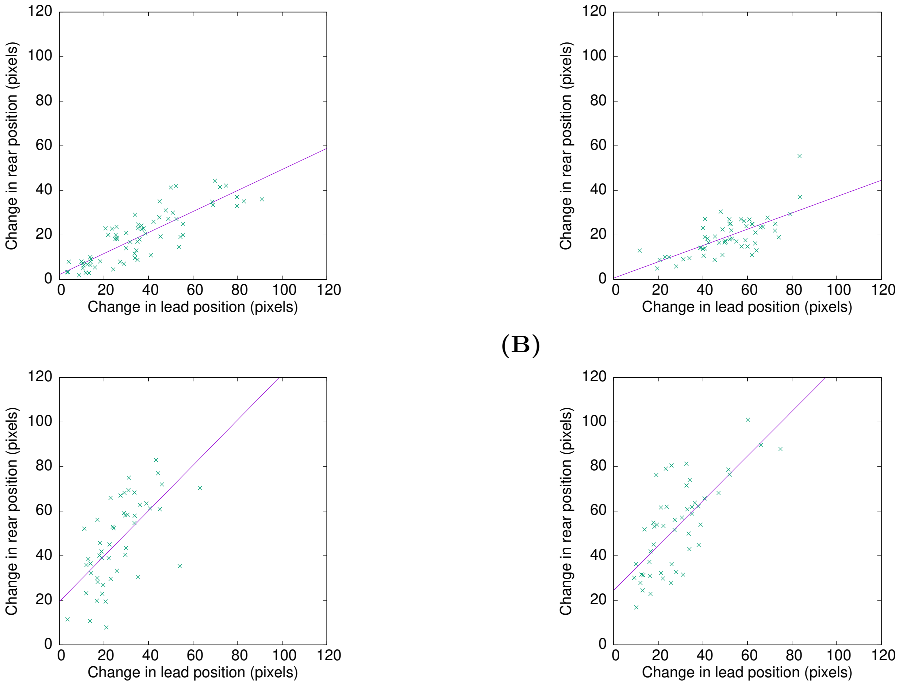

(A) **(B)** **(C)** **(D)**

Fig D.2. These figures show the change in the rear position, ΔR, of each nucleus against the change in leading position, ΔF, of the same nucleus. Each point in each of the graphs represents one nucleus. A. shows the changes as the nucleus moves from before the constriction to beginning to enter the constriction. B. shows the changes from when the nucleus is entering the constriction, to when it is fully in the constriction. C. shows when the nuclei are moving from in the constriction to leaving the constriction. Finally, D. shows the nuclei as they go from leaving the constriction to having fully exited the constriction. The lines of best fit, $y = mx + c$, in each image are for A. $y = 0.47(\pm 0.04)x + (2.23 \pm 1.48)$, for B. $y = 0.37(\pm 0.05)x + 0.68(\pm 2.81)$, for C. $y = 1.02(\pm 0.18)x + 19.4014(\pm 5.179)$ and for D. $y = 1.00(\pm 0.1375)x + 24.52(\pm 4.38)$.

Fig D.2 shows the changes in position of the rear of each nucleus, against the change in position of the front of the same nucleus, with a linear fit of the form $y = mx + c$. The gradient, m, of the lines of best fit, as shown in each of the graphs, provides an estimate of the how far the front position of the nucleus will move, given a change in the rear position, or vice versa. The intercept with the y axis measures how much the rear of the nucleus will move when the front of the nucleus does not change position. The y intercept is near zero in Figs D.2A and D.2B, consistent with the nucleus being unable to move the rear without the front of the nucleus deforming as it entering the constriction, as the out of plane direction is already filled by the nuclei volume. In Figs D.2C and D.2D, the y intercept is larger than zero, representing the nucleus filling the volume in the out of plane direction and, unlike the entry position, the nucleus can move freely into the larger space post-constriction.

The gradient, m, provides an estimate of where the point of zero deformation should be between each average deformation of the nucleus. The nuclei are initially aligned by

the centres of mass, and then shifted an amount along the x axis, to reflect the change given by the ratio of the change in position of the rear to the change in position of the front of the nuclei. As the nuclei are orientated so that they all move in the positive x direction, the value of the ratio is always positive.

If the ratio of the change in the rear position to the change in the front position, m, is in the range $0 \leq m \leq 1$, then the the front moved more than the rear. In this case the area centroid alignment shifts the target too far forward. This is because the front portion of the nucleus is in the constriction which is smaller in the z direction than the back of the nucleus in the larger part of the channel. To correct for this we shift the nucleus backwards by a fraction of the centroid aligned change in rear position, $|\Delta r|$. Shifting it by the full amount $|\Delta r|$ corresponds to the rear of the nucleus being the point of zero deformation. If the ratio of the change in the rear position to the change in the front position is $m \geq 1$, then the rear moved more than the front. In this case the area centroid alignment shifts the target too far back because the front portion of the nucleus is in the larger part of the channel whereas the back is in the smaller constriction. Therefore we shift the target nucleus shape forwards along x relative to the undeformed nucleus by a fraction of the centroid aligned change in front position, $|\Delta f|$. Shifting it by the full amount $|\Delta f|$ corresponds to the front of the nucleus being the point of zero deformation.

The distance that we shift the entire target shape along x is proportional to the relative change between the front and rear position (i.e. the deformation) compared to the sum of the changes to the rear and the front in the respective directions. The proportion of the centroid aligned changes in rear/front position, $|\Delta r|$ or $|\Delta f|$, that the target is shifted to align with the estimated translation free position is,

$$\frac{\Delta R - \Delta F}{\Delta R + \Delta F} = \frac{m-1}{m+1}, \tag{9}$$

where ΔF and ΔR and changes in the front/rear between the undeformed and deformed shapes and Δf and Δr are the changes in the front/rear between the undeformed and deformed shapes when aligned in the centroid frames respectively. Note that in the case of Figs D.2C and D.2D the nonzero y intercept, c, is not included in the calculation of the left hand side of equation 9 such that this equation is valid for all cases. The non zero c in these cases is the part of the deformation that is independent of the translation. It therefore does not contribute to the correction of the centroid alignment to the translation free alignment. The values used to shift each target shape are given in table D.1.

E Strain energy

We plot the free energy density per unit volume, Equation (3) in the main text, against the arclength for the solid and shell models in Figs E.3 and E.4 respectively.

Position	Gradient, m	Shift of target nucleus position from centroid
Before to entering	0.47	$-0.36\lvert\Delta r\rvert = -1.7$
Entering to in	0.37	$-0.46\lvert\Delta r\rvert = -7.3$
In to exiting	1.02	$0.01\lvert\Delta f\rvert = 0.15$
Exiting to out	1.00	$0.00\lvert\Delta f\rvert = 0.0$

Table D.1. The changes in position used to shift the target shape. Δr and Δf are the changes between the rear and front position of the nuclei when aligned in the centroid frames respectively. All distances are in pixels.

F Example of nuclear failure to pass through the constriction

Fig F.5 shows the deformation and traction force fields and actin intensity for a nucleus as it attempts, but fails, to enter the constriction (for a single example cell). Whilst the majority of cells pass through the constrictions, some do not. As discussed in the main text cells treated to disrupt Arp2/3 actin polymerisation are less likely to pass through the constriction. For the full details of the percentages of cells that pass through constrictions of different sizes and with different treatments, see [3].

References

1. Schneider CA, Rasband WS, Eliceiri KW. NIH Image to ImageJ: 25 years of image analysis. Nat Meth. 2012;9:671–675. doi:http://dx.doi.org/10.1038/nmeth.2089.

2. Subtract background: ImageJ Documentation Wiki;. Available from: `http://imagejdocu.tudor.lu/doku.php?id=gui:process:subtract_background`.

3. Thiam HR, Vargas P, Carpi N, Crespo CL, Raab M, Terriac E, et al. Perinuclear Arp2/3-driven actin polymerization enables nuclear deformation to facilitate cell migration through complex environments. Nature communications. 2016;7:10997. doi:10.1038/ncomms10997.

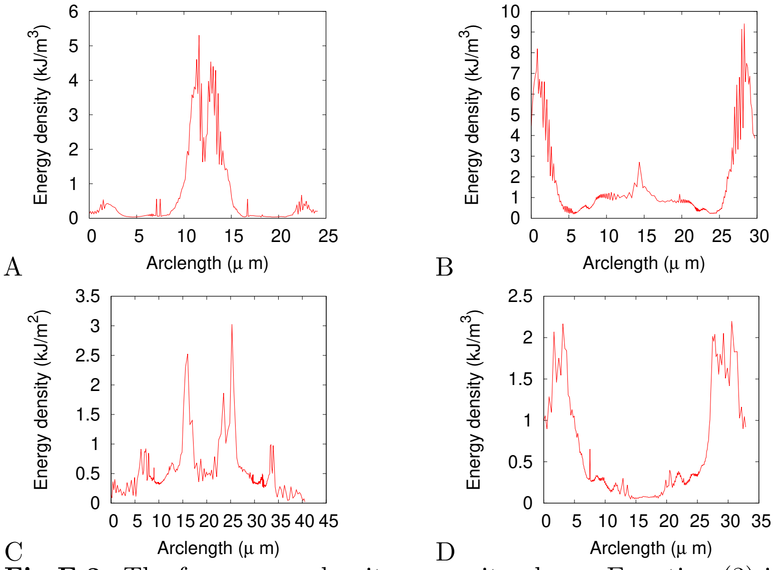

Fig E.3. The free energy density per unit volume, Equation (3) in the main text, for the solid model against the arclength in micrometers. The energy density is given in units of kJ/m^3, which is equivalent to fJ/μm^3. The direction of the arclength coordinate s_1 is anticlockwise as shown in Fig 3 in the main text. $s_1 = 0$ corresponds to $\theta = -\pi$ and $y = 0$ at the rear of the nucleus (x most negative) in Fig 4 in the main text. s_1 then increases anticlockwise around the negative y region to the front (x most positive) then around the positive y region to rear again. We use a Young's modulus of $E = 5\,$kPa and assume that the nucleus behaves as an incompressible elastic solid with a Poisson ratio $\nu = 0.5$. The subfigures correspond to the same times as in Fig 4 in the main text, i.e. A. before to entering constriction, B. entering to within constriction, C. within to exiting constriction and D. exiting to after constriction.

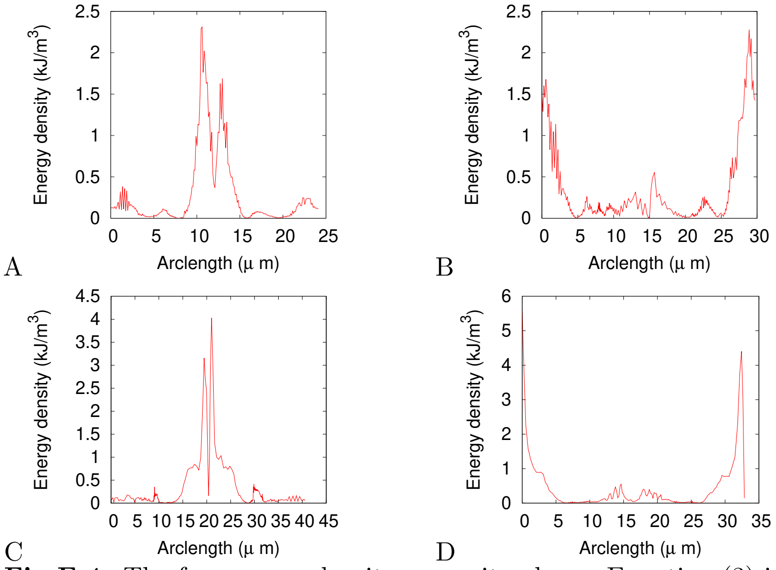

Fig E.4. The free energy density per unit volume, Equation (3) in the main text, for the shell model against the arclength in micrometers. The energy density is given in units of $\mathrm{kJ/m^3}$, which is equivalent to $\mathrm{fJ}/\mu\mathrm{m}^3$. The direction of the arclength coordinate s_1 is anticlockwise as shown in Fig 3 in the main text. $s_1 = 0$ corresponds to $\theta = -\pi$ and $y = 0$ at the rear of the nucleus (x most negative) in Fig 4 in the main text. s_1 then increases anticlockwise around the negative y region to the front (x most positive) then around the positive y region to rear again. We use a Young's modulus of $E = 5\,\mathrm{kPa}$ and assume that the nucleus behaves as an incompressible elastic solid with a Poisson ratio $\nu = 0.5$. The subfigures correspond to the same times as in Fig 6 in the main text, i.e. A. before to entering constriction, B. entering to within constriction, C. within to exiting constriction and D. exiting to after constriction.

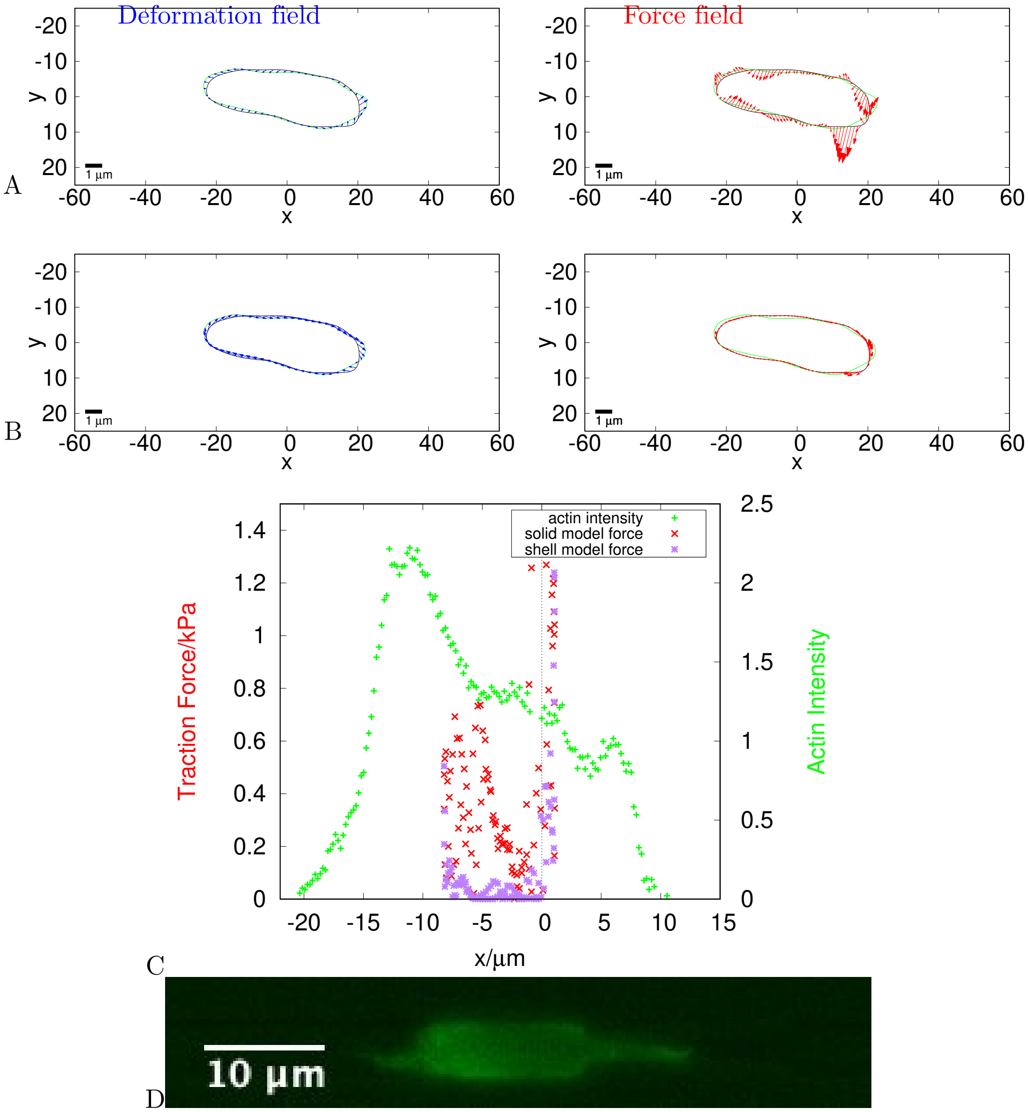

Fig F.5. Deformation (left) and force (right) fields for A. the solid model and B. the shell model for an example nucleus that fails to pass through the constriction. The initial shape is before and the target shape is when the nucleus is attempting to enter the constriction. After this time point the cell changes direction and moves back along the channel in the negative x direction. As in Fig 4 in the main text, the axes show pixel numbers where each pixel is is 0.215μm. The black outline is the initial shape and the green outline is the target deformed shape. Blue arrows represent the final deformation field found between the images and red arrows represent the traction force direction and magnitude, with each arrow scaled such that one unit of length on the axes represents a traction force of 250 Pa. The traction force is calculated using a Young's modulus of $E = 5$ kPa and assuming that the nucleus behaves as an incompressible elastic solid with a Poisson ratio $\nu = 0.5$. C. Average actin (LifeAct-GFP fluorescence) intensity (green + points) at the time point when the nucleus is attempting to enter into the constriction (right hand y-axis). The actin intensity is the mean intensity over the width of the channel at each pixel position. This is then renormalised by the average intensity for the cell and aligned with the start of the constriction at $x = 0$. The red $\times$ points are the absolute values of the y components of the traction force from the solid model A. and the purple $\ast$ points are the absolute values of the y components of the traction force from shell model B. The vertical dotted line indicates the start of the constriction. D. Experimental image showing the actin intensity.
